# Synthesis and stability studies of bicyclo[6.1.0]nonyne scaffolds for automated solid-phase oligonucleotide synthesis†

**DOI:** 10.1039/d3ra08732h

**Published:** 2024-05-29

**Authors:** Kristina Karalė, Martin Bollmark, Antanas Karalius, Mónica Lopes, Oswaldo Pérez, Roger Strömberg, Ulf Tedebark

**Affiliations:** a Department of Biosciences and Nutrition, Karolinska Institutet Neo 141 57 Huddinge Sweden kristina.karale@ri.se; b RISE, Department Chemical Process and Pharmaceutical Development Forskargatan 18 SE-15136 Södertälje Sweden ulf.tedebark@ri.se; c School of Chemistry, University of Southampton Southampton UK; d Faculty of Pharmaceutical Sciences, University of Iceland Sæmundargata 2 102 Reykjavík Iceland; e Department of Laboratory Medicine, Karolinska Institutet ANA Futura 141 52 Huddinge Sweden

## Abstract

Two novel bicyclo[6.1.0]nonyne (BCN) linker derivatives, which can be directly incorporated into oligonucleotide sequences during standard automated solid-phase synthesis, are reported. Stabilities of BCN-carbinol and two BCN-oligonucleotides are evaluated under acidic conditions. In addition, derivatized BCN linkers (non-acidic and acid treated) are evaluated for strain-promoted alkyne–azide cycloaddition (SPAAC).

## Abstract 2

Synthesis and stability studies of BCN ON scaffolds used in SPAAC conjugations.

## Graphical abstract

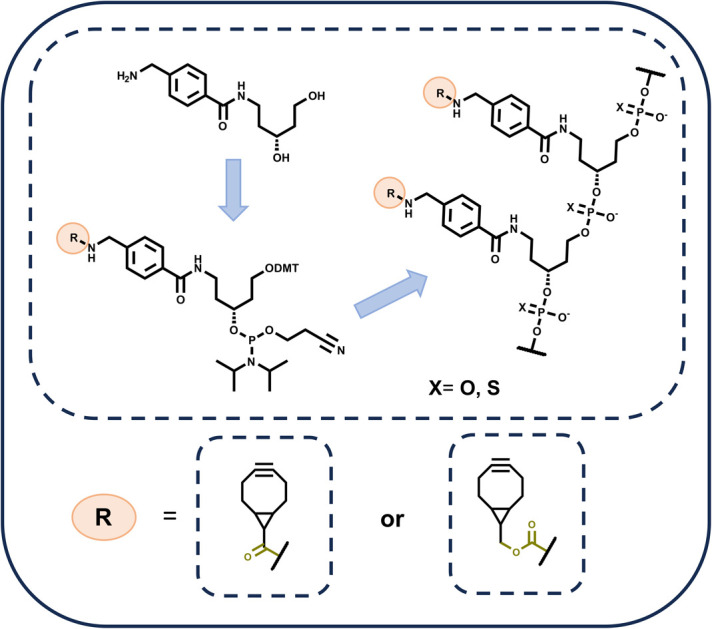

## Introduction

Oligonucleotide (ON) conjugates are an important subgroup of ON therapeutics. The conjugation strategy holds capabilities for improving several characteristics of ON, therefore oligonucleotide conjugates are a promising subset of candidates for clinical use.^1–3^ For example, the conjugation of ONs with various ligands can provide or improve properties such as cell and/or tissue targeting, cellular internalization, as well as pharmacokinetics. In addition, multiple functionalization of the ONs can further furnish ONs and provide additional tailored properties.^4^ Commonly, extra synthetic steps and/or post-ON assembly is needed to prepare ON multi-conjugates.^5^ A handful of approaches have been developed for the preparation of ON multi-conjugates using different types of strategies.^5–10^ However, conventional post-synthetic labeling protocols, *e.g.* amide bond formation, often give low yields, and require prolonged reaction times and high concentration of biomolecule and coupling agents. Copper-catalyzed cycloaddition of alkynes and azides (‘click’ reaction) is a favorable alternative.^11–13^ However, despite its potential for bioconjugation, its usage to prepare ON-conjugates is somewhat limited as copper can cause an undesirable strand degradation of DNA,^14,15^ protein denaturation,^16^ difficulties in product purification^17–19^ and potential problems in conjugation of phosphorothioate ONs (although the latter has been reported for conjugation on a solid support^20^).

On the other hand, strain-promoted alkyne–azide cycloaddition (SPAAC) was employed by several research groups as a copper-free alternative. Commercially available examples of such alkynes are dibenzoazooctyne (DBCO) and non-benzoannulated bicyclo[6.1.0]nonyne (BCN) derivatives. Even though DBCO is widely used, properties, such as high lipophilicity and the tendency to form a mixture of regioisomeric adducts, which is common to all dibenzofused cyclooctynes, limits the attractiveness of this type of click handle. As an alternative to dibenzofused cyclooctynes, BCN was introduced by van Delft *et al.*^21^ and an extensive number of studies were performed by the same group to enable BCN for biorthogonal labeling.^2,18,21–23^ On the other hand, the same authors assessed that BCN is incompatible with standard automated ON synthesis conditions (prolonged/repetitive acid treatment) which limits the versatility of the moiety^2^ and potentially is excluding BCN moieties from introduction in the 3′ end. It has also been reported that strained alkynes in BCN conjugates are labile to acidic conditions and prone to form inactive vinyl alcohol and ketone species.^21^

Compared to DBCO, BCN provides lower lipophilicity as well as a plane of symmetry which prevents the formation of regioisomers. While carbamates remain the most common and commercially available BCN derivatives, a few approaches were developed to equip BCN carbinol with other relevant functionalities.^22–28^ For example, an intriguing alternative to use BCN carboxylic acid as a reactive handle instead of BCN carbinol was quite recently presented by Rady *et al.*^29^ The stabilities of BCN amide and BCN carbamate were compared in various media. The results showed that BCN linked with a carbamate is less suitable for biological applications where prolonged incubation is required but BCN linked with an amide could be used instead.^29^ On the other hand, the less stable carbamate may be desirable in certain circumstances, *e.g.* prodrug design.^30^

The possibility to employ BCN derivatives directly in ON manufacturing seemed appealing to us even after having considered the reported limitations of BCN towards acidic treatments. There are commercially available BCN derivatives in phosphoramidite form, however only for terminal introduction, whereas we suggest BCN-phosphoramidite derivatives with a removable protecting group suitable for incorporation in ON sequences in multiple positions or prior to introduction of additional amidites. There are limitations for copper catalyzed click reactions of phosphorothioate ONs (at least in solution) and P(S) is the most common modification. Thus, fully P(S) containing ONs would be the best models for the two reported novel BCN-functionalized linkers (carbamate and amide based).

Stability studies of the BCN carbinol and BCN carboxylic acid derivatives were performed under acidic conditions typically used during oligonucleotide synthesis. In this study we intended to explore a procedure that allows the direct incorporation of multiple linker units into oligonucleotide phosphorothioates during automated solid phase ON synthesis and the further conjugation of the ON without post assembly derivatization. The ability of both linkers to participate in SPAAC reactions is demonstrated *via* conjugations with an azide derivative.

## Results and discussion

### Preparation of BCN linker derivatives

**Fig. 1:**
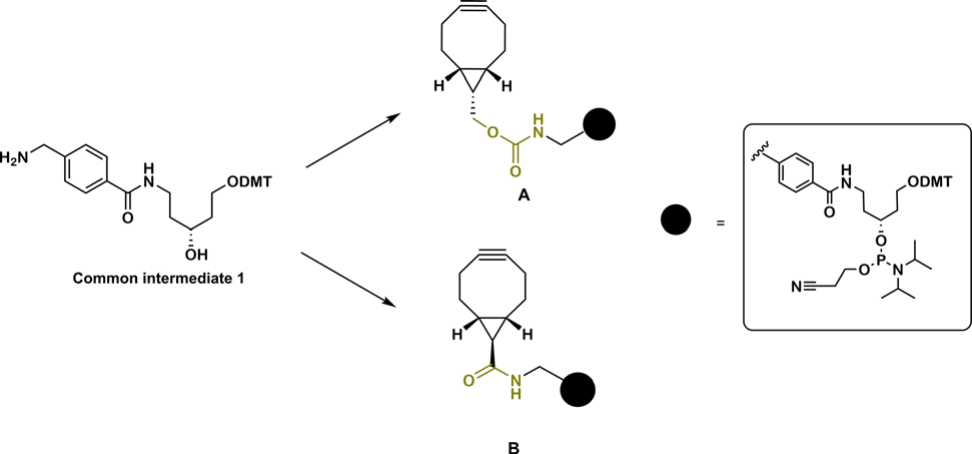
BCN functionalized linkers A and B for solid phase oligonucleotide synthesis.

The design of BCN-derivatized linkers A and B (Fig. 1) contains previously reported scaffold (1).^7^ In contrast to commercially available BCN phosphoramidites, which allow only terminal modification, this scaffold can provide the possibility to incorporate the linkers as amidite-monomers into multiple positions (and/or allow further extension with other amidites) of an oligonucleotide sequence directly on an oligonucleotide synthesizer. In addition, using 1 as amidite allows the possibility to introduce additional handles for multiple and/or orthogonal conjugation due to the presence of the removable DMT protecting group of the linear aminodiol derivative. As carbamates represent the most encountered type of BCN derivatives and are used in prodrug design,^29^ we initially chose to attach the scaffold 1 to the BCN carbinol *via* a carbamate linkage (Fig. 1, A). The second linker B was designed to contain an amide bond between BCN carboxylic acid and 1 (Fig. 1, B). Rady *et al.* demonstrated that the presence of a carboxylic acid on the BCN scaffold does not change alkyne reactivity and gives a straightforward approach to derivatize BCN with an amine of choice *via* amide formation.^29^ It was also shown that BCN amide is a better alternative for molecular probe design which require hydrolytic stability in biological media.^29^

The synthesis of both linkers A and B started with the preparation of a common intermediate dimethoxytrityl-5*N*-[(methyl)-benzoyl]-aminopentane-1,3-diol 1 (Fig. 1 and ESI Fig. S1†). BCN carbinol was prepared from readily available starting materials using published procedures.^31,32^

**Scheme 1:**
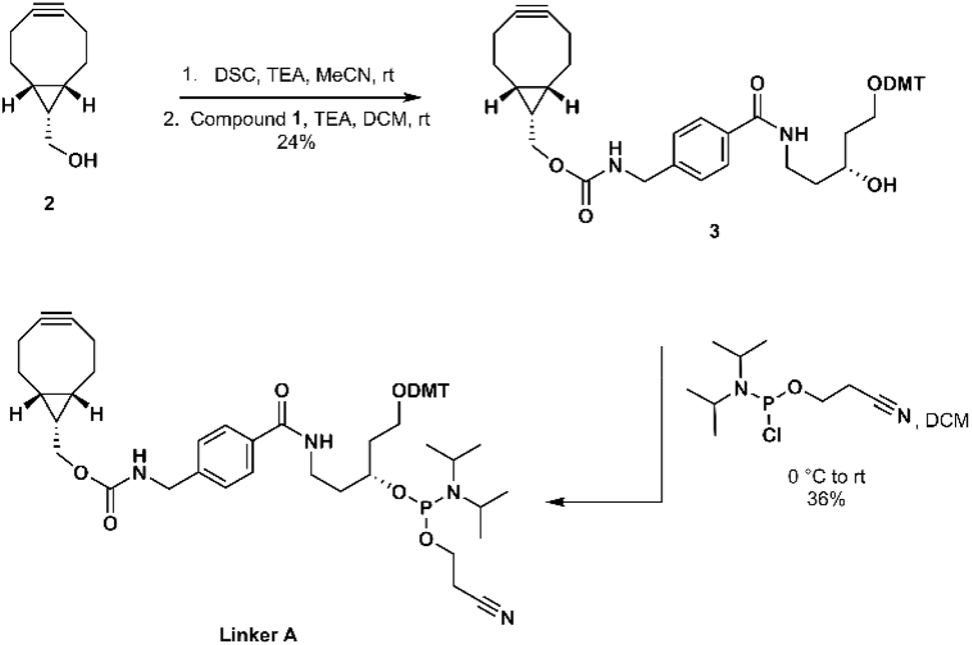
Synthesis of linker A.

The synthesis of linker A was started by activation of BCN carbinol (2) with disuccinimidyl carbonate.^28^ The obtained intermediate was then reacted with 4,4′-dimethoxytritylated compound 1 to give carbamate derivative 3 which was then converted to linker A in amidite form (Scheme 1).

**Scheme 2:**
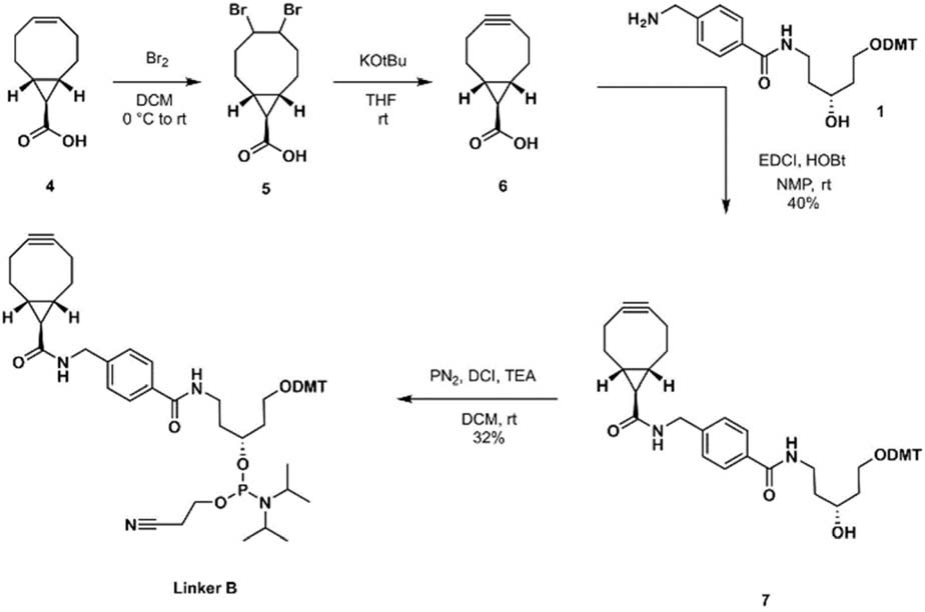
Synthesis of linker B.

Linker B was prepared using BCN carboxylic acid (6) as an intermediate. BCN carboxylic acid 6 was prepared by combining two reported procedures by Rady *et al.*^29^ and by O'Brien *et al.*^32^ The obtained carboxylic acid intermediate 4 (alkene,^32^) was brominated^29^ to 5 and used crude for the alkyne 6 formation. The purity of obtained crude 6 was satisfactory enough to continue for the next step and intermediate 6 was coupled with scaffold 1 using EDCI/HOBt to obtain the amide derivative 7 which, after purification, was converted to linker B in amidite form (Scheme 2).

### Stability investigation of the BCN moiety

As mentioned above, there are some reports in the literature^2^ about the instability of the alkyne functionality of BCN in acidic conditions, which would potentially be incompatible with solid-phase oligonucleotide synthesis other than as a final/terminal addition at the 5′ end. In addition, we have discovered that BCN derivatives obtained in crystalline form and stored in the dark at −10 °C reveal different solubility properties over time suggesting *e.g.*, degradation or other structural modification.

**Fig. 2:**
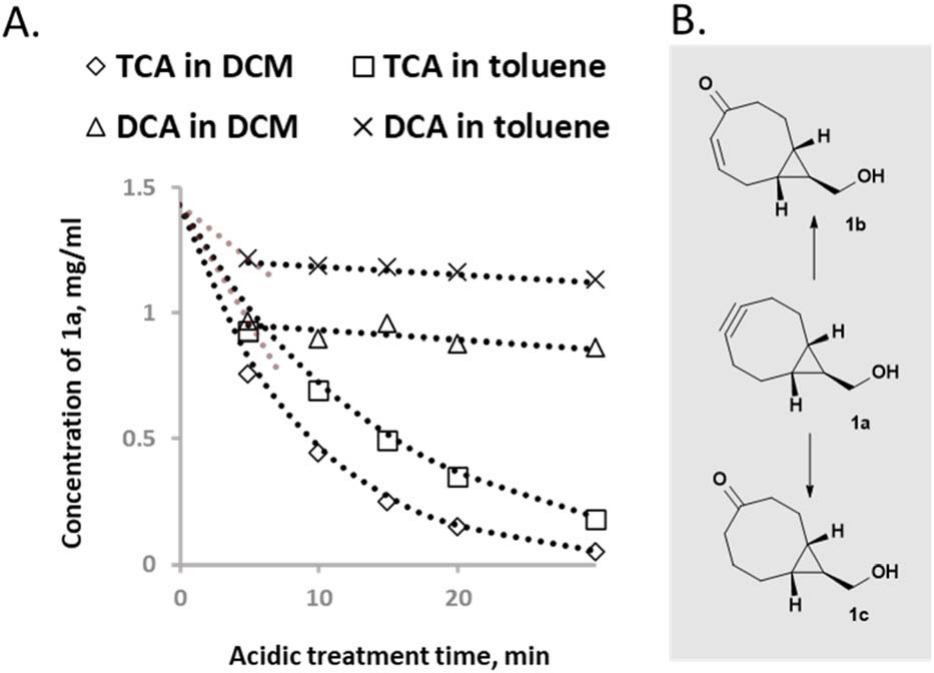
Trend analysis of the stability of BCN carbinol. (A) Kinetic study of BCN carbinol degradation in the presence of solutions of TCA or DCA in DCM or toluene respectively using GC analysis. (B) Degradation products of BCN carbinol, identified by NMR 1b-acid induced hydrolysis and oxidation product (ESI Fig. S19†). 1c-acid induced hydrolysis product.^21^

When scaling up, we noticed that the yields of BCN carbinol were varying and were usually lower than anticipated with regard to reported yields in literature.^32^ Despite having the chromatography columns pre-treated with 0.1–1% TEA to neutralize any acidic groups, the yields of isolated BCN carbinol derivatives were rather low. Therefore, one possible reason for this problem could be the silica used for chromatographic purification. To explore this further, BCN carbinol, as the most studied member of BCN analogs, was chosen as a model compound to evaluate the stability of the alkyne functionality. First, BCN carbinol was evaluated by thin layer chromatography (TLC, ESI Fig S14†) to reveal if degradation occurs over time on TLC silica under ambient conditions. To investigate in more detail, the obtained degradation product was isolated. NMR studies revealed that degradation on TLC plate occurred due to both oxidation and hydrolysis (Fig. 2B, 1b). When using oligonucleotide synthesis conditions (under nitrogen, see below), only alkyne hydrolysis (Fig. 2B, 1c) was detected.

### Stability study on the BCN carbinol

As BCN carbinol was not stable when evaluated on TLC, a series of stability experiments in solution were performed to evaluate the sensitivity of the alkyne functionality in BCN carbinol with a goal to obtain a degradation trend and to assess if it could be compatible with conditions in an automated oligonucleotide synthesizer. TCA (trichloroacetic acid) in DCM or DCA (dichloroacetic acid) in toluene are standard treatments for DMT (dimethoxytrityl) removal. Therefore, samples containing 1.4 mg ml^−1^ BCN carbinol, 3% (w/v) TCA or DCA in DCM or toluene were prepared (Fig. 2, left). Reaction was monitored using GC and samples were analyzed after 5, 10, 15, 20 and 30 min.

The results (Fig. 2) showed that the BCN carbinol is more prone to degradation in the presence of TCA in either toluene or DCM corroborating the results reported by Gibson *et al.*^2^ Though BCN carbinol was marginally more stable in TCA–toluene solution (13% of BCN carbinol remained unaffected compared to 3.5% of unaffected BCN carbinol in TCA–DCM solution), the investigation revealed that DCA is a more suitable detritylating option for BCN derivatives since after 30 min there was 60% of BCN carbinol remaining in DCA–DCM solution and 79% in DCA–toluene solution (Fig. 2 and ESI Fig. S13†). In addition, the decomposition of BCN carbinol in the presence of TCA appears to follow first order kinetics with a reaction rate constant *k* = 0.11 s^−1^ in DCM and *k* = 0.068 s^−1^ in toluene. In the presence of DCA decomposition does not follow such a trend – after an initial drop, slow degradation proceeds in both DCA–DCM and DCA–toluene solutions suggesting that the stability can be acceptable for shorter acidic treatments with only low amounts of degradation. These results also indicate introduction of one or a few BCN scaffolds only at the 5′ end during ON synthesis to minimize the acid degradations.

### Evaluation of BCN-ON derivatives on an oligonucleotide synthesizer

In order to explore the suitability of both linkers for solid phase oligonucleotide synthesis, two model compounds, all-phosphorothioate heptamers (A)_2_-T_5_ and (B)_2_-T_5_ were prepared using standard solid-phase oligonucleotide synthesis conditions without removal of the final DMT protecting group. LC-MS analysis of the linker A and B derivatized oligonucleotides indicated the formation of both heptamers ((A)_2_-T_5_ and (B)_2_-T_5_) as major products (ESI Fig. S16 and S22†).

The study was continued by further evaluation of the stability of the BCN moiety incorporated in the ON sequence using 3% DCA solution in toluene, which is a standard condition for dimethoxytrityl removal during solid-phase oligonucleotide synthesis. A column loaded with solid-support bound (A)_2_-T_5_ or (B)_2_-T_5_ was connected to the oligonucleotide synthesizer. The solid-support bound heptamer derivatives were exposed to five detritylation cycles each. After each acid cycle, 10 mg of solid-supported material was removed for LC-MS analysis.

**Fig. 3:**
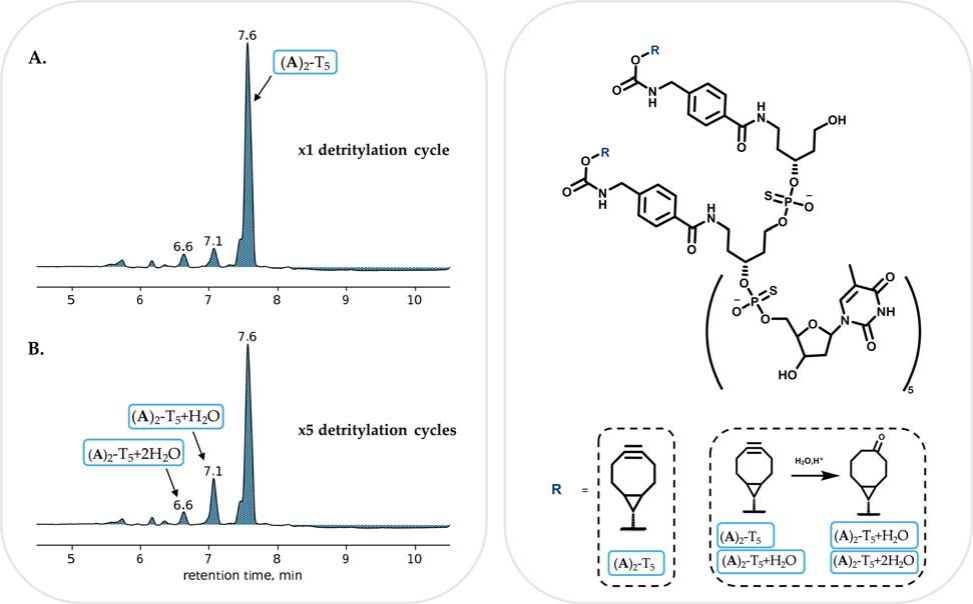
RP-HPLC profile of (A)_2_-T_5_ after one (A) and five (B) detritylation cycles. 7.6 min: (A)_2_-T_5_, 7.1 min (A)_2_-T_5_ + H_2_O, 6.6 min: (A)_2_-T_5_ + 2H_2_O.

In both cases (A)_2_-T_5_ and (B)_2_-T_5_, only minor degradation even after five detritylation cycles could be found (Fig. 3, ESI S19 and S26†). Analyses of both derivatives showed a major product with the masses corresponding to (A)_2_-T_5_ and (B)_2_-T_5_.

BCN carbamate or amide derivatives revealed a similar stability (ESI Fig. S19† (BCN carbamate) and S26 (BCN amide)). In case of the (A)_2_-T_5_ synthesis, traces of (A)_2_-T_5_ + H_2_O and (A)_2_-T_5_ + 2H_2_O could be detected (at 7.1 min and 6.6 min respectively) even after one detritylation cycle (Fig. 3A). After five detritylation cycles this peak has increased (Fig. 3B), with the unaffected BCN derivative remaining the major product. A similar picture could be seen for the (B)_2_-T_5_ as traces of (B)_2_-T_5_ + H_2_O and (B)_2_-T_5_ + 2H_2_O were identified after the first detritylation cycle following the increase of the peaks after five cycles (ESI Fig. S24†).

**Fig. 4:**
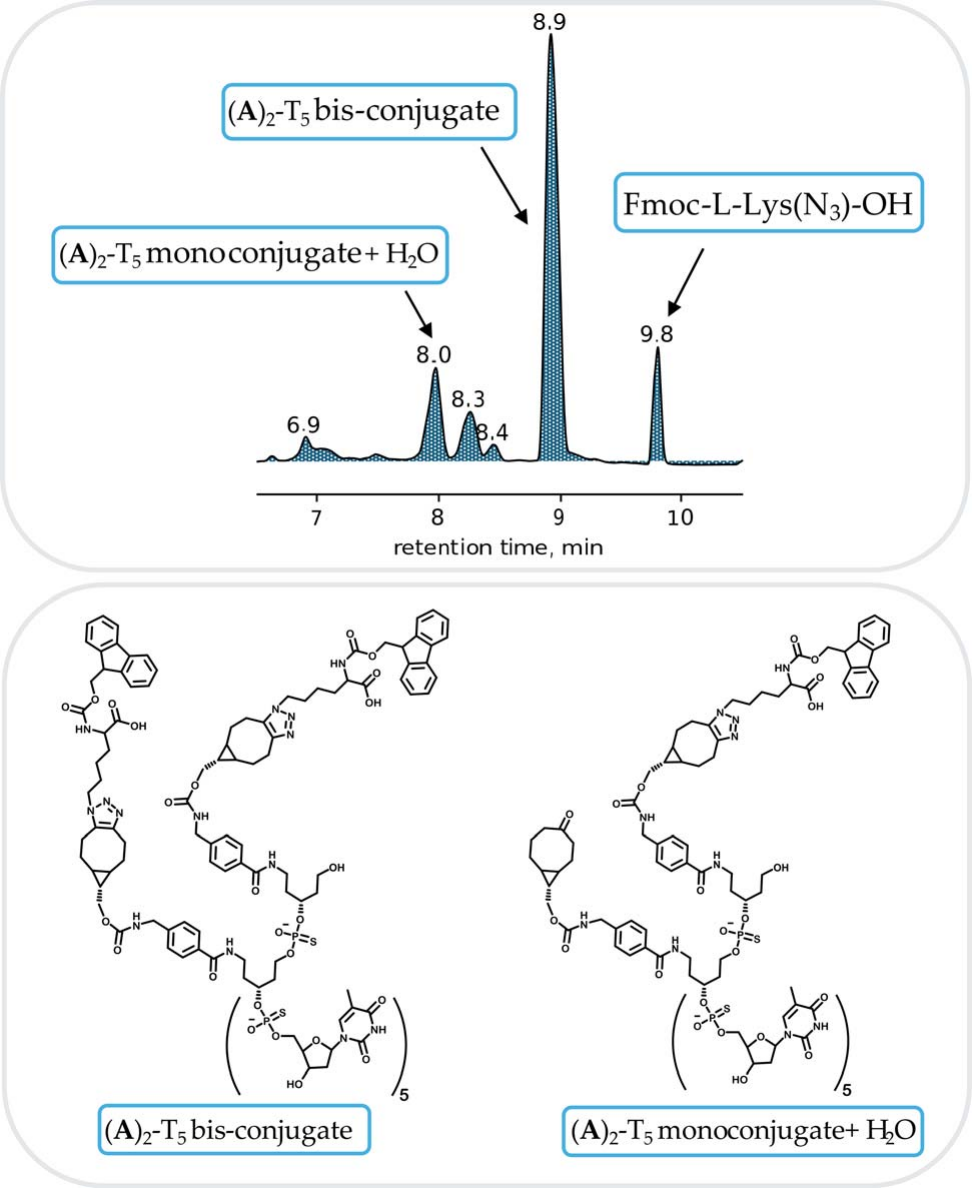
RP-HPLC profile of (A)_2_-T_5_ bis-conjugate. The conjugation reaction was performed overnight ((A)_2_-T_5_ previously treated with five detritylation cycles). 8.9 min: (A)_2_-T_5_ bis-conjugate. 8.4 and 8.3 min: (A)_2_-T_5_ monoconjugate. 8.0 min: (A)_2_-T_5_ monoconjugate + H_2_O (see also text below).

Next, diBCN containing heptamers, (A)_2_-T_5_ and (B)_2_-T_5_ were evaluated for SPAAC. As a model compound, an excess of Fmoc-l-Lys(N_3_)-OH was added to LC vials containing BCN derivatives (A)_2_-T_5_ and (B)_2_-T_5_ in aqueous solution of 8.6 mM TEA + 100 mM HFIP (buffer A) and the reaction was followed by LC-MS (ESI Fig. S17 and S22†). As additional proof that the triple bond to a large extent remained intact after five cycles of detritylation treatments, both (A)_2_-T_5_ and (B)_2_-T_5_ were again reacted with azide-containing Fmoc-l-Lys(N_3_)-OH (Fig. 4) to give the desired bis-conjugates. The remaining intact BCN derivatives (A)_2_-T_5_ and (B)_2_-T_5_ underwent nearly quantitative conversion into the expected triazole adduct (ESI Fig. S20 and S27†).

In Fig. 4 the major peak (8.9 min) corresponds to the formation of the desired bis-conjugate. Traces of monoconjugate (not fully reacted starting compound still containing alkyne) is also detected at 8.3 min. The mass of the peak at 8.0 min (ESI Fig. S20D†) corresponds to monoconjugated derivative containing one hydrolyzed BCN fragment (+H_2_O).

**Fig. 5:**
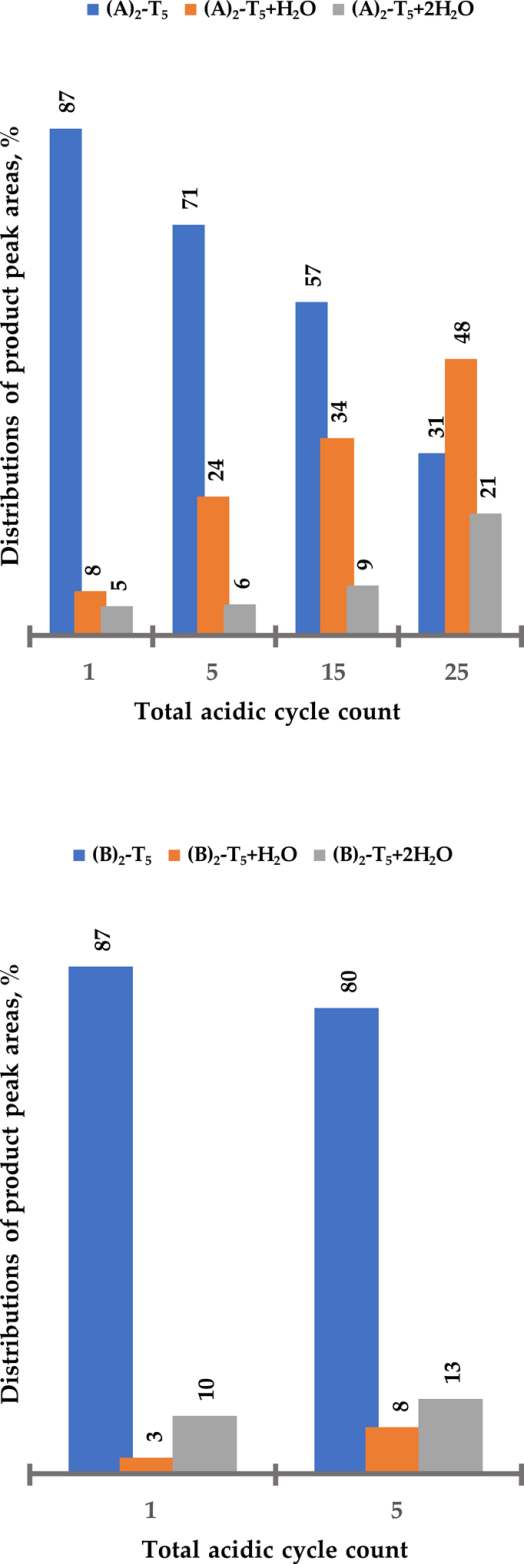
Distribution of (A)_2_-T_5_ and side products ((A)_2_-T_5_ + H_2_O and (A)_2_-T_5_ + 2H_2_O) after increasing number of standard detritylation treatments in percent of total peak area. Increased number of cycles also indicated additional degradation due to a noisier base line (*cf.* ESI Fig. S33†).

To further determine if the BCN linker can be considered as a functionality at the 3′ end, *i.e.*, survive additional 20–25 acidic cycles, both solid-support bound heptamers ((A)_2_-T_5_ and (B)_2_-T_5_ previously treated with five acidic cycles) were exposed to an additional 20 detritylation cycles. Although in this case the goal was to not to compare carbamate *vs.* amide, rather than to find the limitations of triple bond stability, both solid-bound heptamers were subjected to additional acidic treatments to determine the trend of acidic degradation. A sample of solid-bound heptamer (10 mg) was taken after 10 additional cycles (after exposure to 15 acidic cycles in total) and then after 20 additional cycles (exposed to 25 cycles in total). Samples were cleaved from solid support and analysed by LC-MS (Fig. 5 and ESI Fig. S28†).

**Fig. 6:**
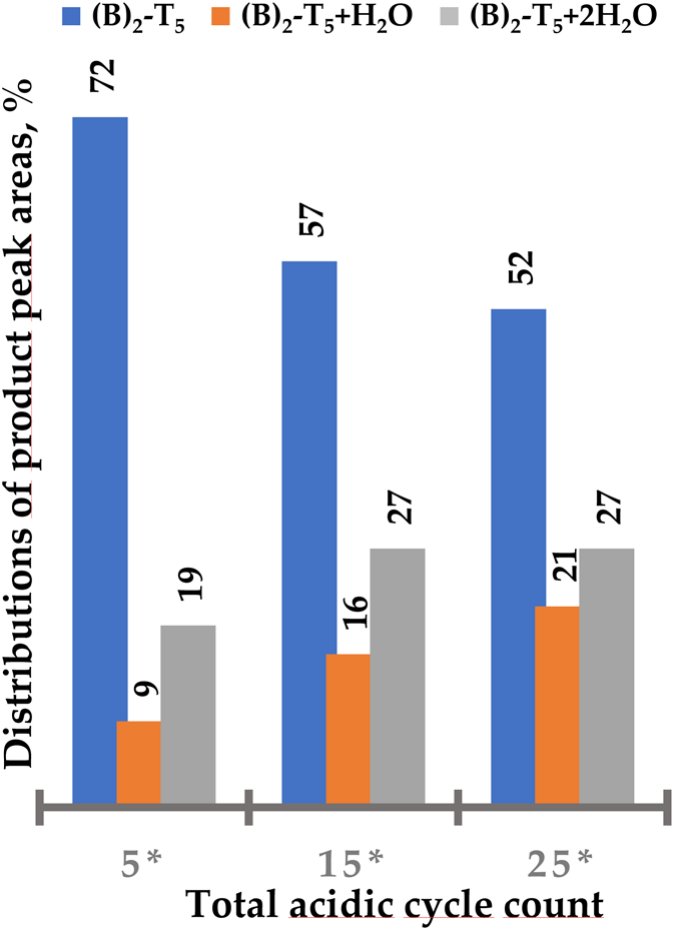
Distribution of (B)_2_-T_5_ and side products ((B)_2_-T_5_ + H_2_O and (B)_2_-T_5_ + 2H_2_O) after increasing number of standard detritylation treatments in percent of total peak area. Increased number of cycles also indicated additional degradation due to a noisier base line (*cf.* ESI Fig. S34†). 5* – the solid support bound (B)_2_-T_5_ after five standard detritylation treatments and stored at ambient temperature for 4 months. Additional 10 and 20 acidic treatments were performed on aged sample, giving in total 15* and 25* acidic treatments (DCA in toluene).

25 detritylation cycles of (A)_2_-T_5_ resulted in substantial degradation of the triple bond (Fig. 5). Approximately 30% of the starting oligomer derivatized with two BCN linkers remained intact, clearly showing that standard detritylation conditions would lead to poor yields of an intact BCN handle in the 3′ position of an ON. (B)_2_-T_5_ proved to be somewhat more stable during 25 detritylation cycles (approximately 50% of starting compound remaining intact, Fig. 6). For an unclear reason, the degradation product when using a BCN amide derivative contains to a higher degree two water molecules whereas the BCN carbamate has to a higher degree one water molecule. Regardless, the use of either BCN derivative in the 3′ position is not considered to be satisfactory enough. Therefore, alternative building blocks containing handles more stable during automated solid-phase oligonucleotide synthesis would be desirable for introduction at the 3′ end.

## Conclusions

This study reports two novel BCN linker amidite derivatives which can be incorporated into oligonucleotide sequences during solid-phase oligonucleotide synthesis. In addition, the BCN linker derivatives are designed to allow sequential addition to the oligonucleotide sequence allowing for multiple conjugation to azide containing compounds, either on solid-phase or in solution (on solid-phase, click reaction could also be performed after each addition and before detritylation as for copper catalyzed reactions). The compatibility with solid-phase conditions was demonstrated by the preparation of two model phosphorothioate oligonucleotides containing the different BCN linkers A and B (Fig. 1). Both BCN containing oligonucleotide derivatives as well as BCN-carbinol were evaluated under different acidic conditions commonly used in oligonucleotide synthesis. Our study revealed that although stability of the BCN moiety may be limited under different acidic conditions, both BCN linker derivatives can readily be incorporated at the 5′ end of a solid-support bound oligonucleotide in at least double copies. Subsequent deprotection and click reactions in solution was also demonstrated to be successful. This is a clear advantage to only terminal addition as well as to copper catalyzed reaction that needs to be performed on solid support.^6^ Both model oligonucleotide derivatives keep their alkyne functionality intact to 70–80% after five acidic cycles. We suggest that this is an acceptable level for introducing multiple BCN labeled linkers (at least two to three) at the 5′ end and then used for the preparation of multi-conjugates. In combination with orthogonal types of linkers, various modalities could then be conjugated. On the other hand, incorporation of BCN handles at the 3′ end or at other positions requiring a larger number of acid treatments seems to lead to a too severe degradation of the alkyne functionality using standard detritylation conditions, and thus quite poor yields of potential conjugates would be expected.

## Author contributions

Conceptualization, K. K., M. B., R. S., U. T.; methodology, K. K., M. B., R. S., U. T.; investigation, K. K., M. B., A. K., M. L., O. P.; resources, R. S. and U. T.; draft manuscript preparation, K. K.; visualization, K. K., A. K. Writing, reviewing, and editing, all authors; supervision, M. B., R. S., U. T.; project administration, U. T.; funding acquisition, K. K., R. S., U. T.; all authors have read and agreed to the published version of the manuscript.

## Conflicts of interest

The authors declare no conflict of interest.

## Supplementary Material

RA-014-D3RA08732H-s001
