# Supplementary material for: Synthesis and stability studies of bicyclo[6.1.0]nonyne scaffolds for automated solid-phase oligonucleotide synthesis
Source: RSC Adv. 2024 May 29;14(25):17406–12. doi: 10.1039/d3ra08732h (PMC11134333; doi:10.1039/d3ra08732h)
Supplement: RA-014-D3RA08732H-s001 [file RA-014-D3RA08732H-s001.pdf]

Electronic Supplementary Material (ESI) for RSC Advances.
This journal is © The Royal Society of Chemistry 2024

Supplementary data

Synthesis and Stability Studies of Bicyclo[6.1.0]nonyne Scaffolds for Automated Solid-Phase Oligonucleotide Synthesis

Kristina Karalė[1,2], Martin Bollmark[2], Antanas Karalius[2], Mónica Lopes[2,3],Oswaldo Pérez[2,4], Roger Strömberg[1,5] and Ulf Tedebark[2,*]

[1]Department of Biosciences and Nutrition, Karolinska Institutet, Neo, 141 57, Huddinge, Sweden; roger.stromberg@ki.se

[2]RISE, Department Chemical Process and Pharmaceutical Development, Forskargatan 18, SE-15136 Södertälje, Sweden; kristina.karale@ri.se (K.K.), martin.bollmark@ri.se (M.B.), oswaldo.perez@ri.se ulf.tedebark@ri.se

[3]School of Chemistry, University of Southampton, Southampton, United Kingdom

[4]Faculty of Pharmaceutical Sciences, University of Iceland, Sæmundargata 2, 102 Reykjavík, Iceland

[5]Department of Laboratory Medicine, Karolinska Institutet, ANA Futura, 141 52, Huddinge, Sweden

* To whom correspondence should be addressed. Tel: +46105166553; Email: ulf.tedebark@ri.se

Contents

S-1. Experimental Section 2

BCN carbinol 3

BCN-linker containing oligonucleotides 3

SPAAC evaluation 3

S-2. NMR spectra of linkers and intermediates 8

S-3. Kinetic studies of BCN carbinol 19

S-4. RP-HPLC profiles and MS analyses of oligomer stability studies 21

S-1. Experimental Section

General Information

Unless otherwise stated, all reagents and solvents used in chemical synthesis were of commercial grade.

HPLC was performed on UltiMate 3000 HPLC system with UV detection at 254 nm.

Chromatographic separations were performed on CombiFlash® Rf 200 by Teledyne ISCO system using Biotage® normal or reversed-phase (RP) silica columns.

LC-MS analyses were performed on Waters Aquity Ultra performance system equipped with an SQ detector or with Thermo Scientific ISQ EM MS system. MS spectra were collected by elution of the oligomer on an Oligonucleotide BEH C18 2.1 x50mm 130 Å 3µm column using 0.4 mL/min gradient from 0-90-95% or from 0-60-80% methanol (MeOH) in 8.6 mM triethylamine (TEA) + 100 mM 1,1,1,3,3,3-hexofluoroisopropanol (HFIP) buffer over 15 min at 40 °C or by elution of the compounds on an Aquity UPLC BEH C18 (2.1 x 50 mm), 1.7 µm column using 0.4 mL/min gradient from 0-95% acetonitrile (MeCN) in 0.1 M NH4OAc buffer over 7 min at 40 °C. UV was detected at 254 nm. Ionization mode: ESI negative. HRMS spectra were collected by elution of the samples on Acquity BEH C18 1.7 µm 100x2.1 column using a 0.4 mL/ min linear gradient from 0 to 95% acetonitrile in water over 7 min at 40 C on Waters Xevo G2-XS QTof. UV was detected at 234 nm. Ionization mode: ESI positive. Source capillary voltage: 3.5 kV. Source desolvation temperature: 500 C.

Phosphorothioate oligonucleotide syntheses were performed using an ÄKTA oligopilot 10 synthesizer and conventional RNA/DNA synthesis procedures on 50 µmol ((A)2-T5) or 25 µmol ((B)2-T5) scales. All reagents, solvents and building block solutions were kept under inert (N2) atmosphere. 0.1 M anhydrous MeCN solutions of commercial T amidite (Thermo Fisher Scientific) and synthesized BCN linker amidites A and B were used. The ON syntheses were performed using Primer Support™ 5G T 350 solid support. 0.3 M 5-benzylthio-1-H-tetrazole (BTT) was used as an activator and 0.1 M xanthane hydride in pyridine as sulfurizing agent. Coupling times were as follows: 2.5 min for DNA phosphoramidites and 12 min for BCN linker phosphoramidites (linker A and linker B).

GC analysis was performed with an Agilent Technologies 6850 GC system.

NMR spectra were recorded using a Bruker AV 500 MHz (500.13 MHz in 1H, 125.76 MHz in 13C and 202.47 MHz in 31P, 470.56 MHz in 19F) spectrometer using the deuterated solvent signal as an internal standard. Chemical shifts (δ scale) are reported in parts per million (ppm). Coupling constants (J values) are given in Hertz (Hz).

Stability studies

BCN carbinol

Four detritylating solutions containing 3% (w/v) trichloroacetic acid (TCA) or 3% (w/v) dichloroacetic acid (DCA) in either dichloromethane (DCM) or toluene were prepared. 10 mg of BCN carbinol and 16 µl dodecane were dissolved in each detritylating solution (7 ml). The reaction was monitored by GC using dodecane as an internal standard. Samples were analyzed after 5 min, 10 min, 15 min, 20 min and 30 min. Before analysis, the reaction was quenched with 2 M (2 equiv.) $KHCO_3$.

BCN-linker containing oligonucleotides

A column loaded with solid-supported all-P(S)-heptamer $(A)_2$-T_5 (50 µmol) or all-P(S)-heptamer $(B)_2$-T_5 (25 µmol) was connected to the oligonucleotide synthesizer. Each derivative was exposed to five detritylation cycles (15 column volumes (CV) of 3% DCA in toluene per cycle, linear flow 400 cm/h). After each acidic cycle, 10 mg of solid-supported material was removed for LC-MS analysis. In addition, after 5 acidic cycles including subsequent washing with MeCN, both oligonucleotide derivatives were evaluated again for SPAAC.

SPAAC evaluation

Fmoc-L-Lys(N3)-OH solution (9 µL, 0.01 M in DMSO) was added to LC vials containing approx. 0.2 mg/mL of heptamer $(A)_2$-T_5 or $(B)_2$-T_5 in water (1 mL) with 8.6 mM TEA + 100 mM HFIP. The reactions were followed by LC-MS.

Chemical syntheses and characterizations

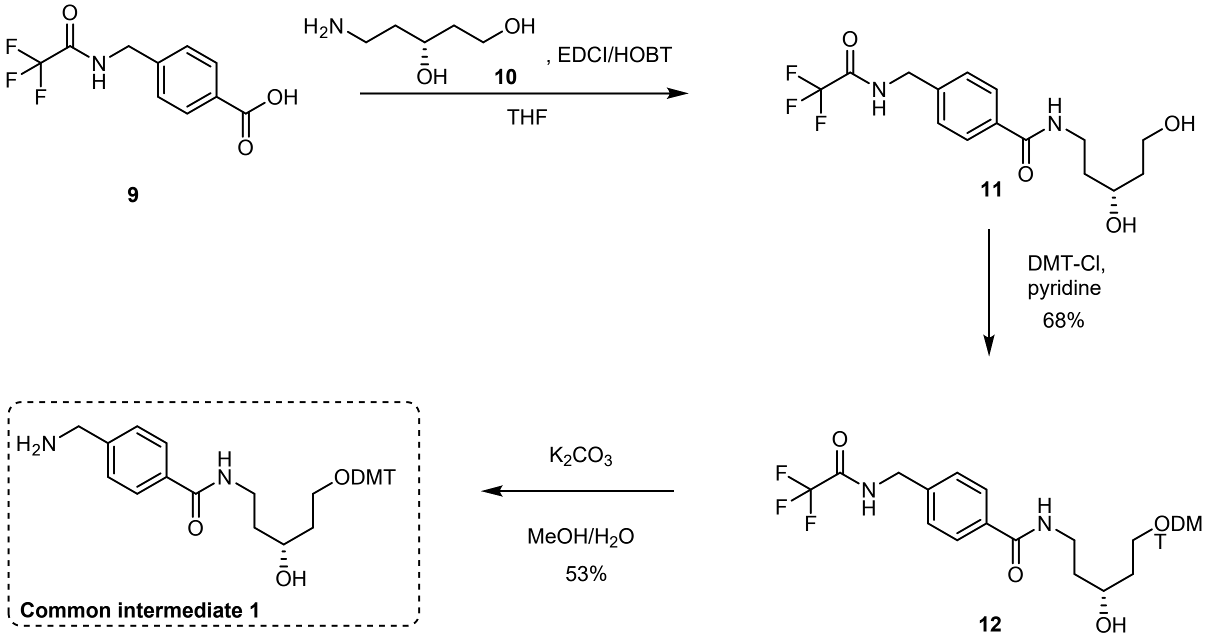

Figure S1. Synthesis route of common intermediate 1.

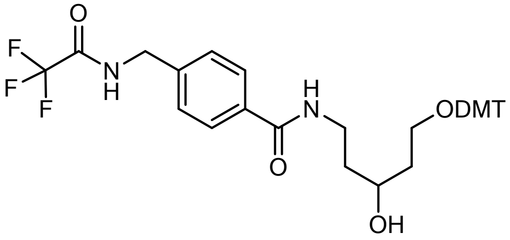

***(R)*-1-O-(4,4′-Dimethoxytrityl)-*5N*-[((2,2,2-trifluoroacetamido)methyl)-benzoyl]-aminopentane-1,3-diol (12).** Aminodiol **10** was prepared according to a published procedure [1]. To a solution of compounds **9** (10.0 g, 40.4 mmol) and **10** (6.75 g, 56.6 mmol) in THF/water (2:3, 50 mL), HOBt (1.1 g, 8.1 mmol) and EDCI (7.5 g, 49 mmol)

A B F C D E

were added, and the reaction mixture was stirred for 2 h. After the completion of the reaction (monitored by RP-HPLC), THF and partially water was evaporated under reduced pressure until precipitation occurred. The crude intermediate **11** was filtered, washed with water, dried to give 9.2 g (65%) as a white powder and used without additional purification. 6.0 g (17 mmol) of **11** were co-evaporated with pyridine and re-dissolved in the same solvent (30 mL). DMT-Cl (6.1 g, 18 mmol) was added under nitrogen atmosphere and the reaction mixture was stirred overnight. Then the reaction was quenched with methanol (2 mL) followed by the addition of 10% aqueous $NaHCO_3$ solution. Volatiles were evaporated under reduced pressure and followed by a water/EtOAc work-up. The organic phase was collected and washed with water and 10% aqueous citric acid solution, dried over Na_2SO_4, filtered, and evaporated to give 7.7 g (68%) of intermediate **12.** ^{1}H NMR (500 MHz, DMSO-d_6) δ 10.04 (t, J = 6.1 Hz, 1H, NH), 8.40 (t, J = 5.6 Hz, 1H, NH), 7.83 – 7.77 (m, 2H), 7.40 – 7.16 (m, 11H), 6.86 (d, J = 6.8 Hz, 4H), 4.47-4.40 (m, 3H, OH+2H, H^A), 3.72 (s, 6H, -OMe), 3.70 – 3.63 (m, 1H, H^D), 3.39-3.27 (m, 2H+H_2O, H^B), 3.05 (t, J = 7.3 Hz, 2H, H^F), 1.79 – 1.57 (m, 3H, $2H^E$+H^C), 1.55-1.45 (m J = 13.4, 7.8 Hz, 1H, H^C).^{13}C NMR (126 MHz, DMSO-d_6) δ 165.9, 157.9, 156.4 (q, $^2J_{CF}$=36 Hz), 149.6, 145.3, 140.4, 136.1, 136.1, 133.8, 129.6, 127.7, 127.6, 127.3, 127.1, 126.5, 118.0,115.9 (q, $^1J_{CF}$=288 Hz), 113.1, 85.2, 65.3, 60.3, 55.0, 42.3, 37.4, 37.2, 36.4. ^{19}F NMR (470.56 MHz, CD_3OD) δ=-77.10 ppm. HRMS calcd. for $C_{36}H_{37}F_3N_2O_6$ $[M+H]^+$ 651.2682, found 651.2672.

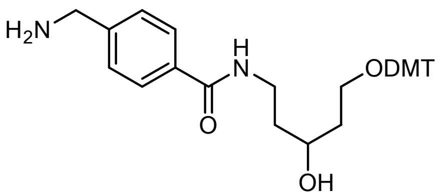

***(R)*-1-O-(4,4′-Dimethoxytrityl)-*5N*-[(methyl)-benzoyl]-aminopentane-1,3-diol (common intermediate 1).** Compound **12** (6.0 g, 9.2 mmol) dissolved in MeOH (30 mL) followed by the addition of water (3 mL) and K_2CO_3 (2.5 g, 18.4 mmol). Reaction mixture stirred overnight at RT. Then a sufficient amount of toluene/water mixture added to get a phase separation. The organic phase was washed with 10% aqueous $NaHCO_3$ solution and concentrated under reduced pressure. Obtained crude material was subjected to RP column and chromatographed using 0 to 100% gradient of MeCN (containing 0.1% TEA) in water (containing 0.1% TEA) to yield compound **1** (2.7 g, 53%) as a white foam. ^{1}H NMR (500 MHz, CD_3OD) δ 7.78 (d, J = 6.5 Hz, 2H), 7.40 (d, J = 18.0 Hz, 4H), 7.29 – 7.20 (m, 6H), 7.17 (d, J = 7.6 Hz, 1H), 6.78 (s, 4H), 3.96 – 3.88 (m, 1H, H^D), 3.84 (s, 2H, H^A), 3.75 (s, 6H, -OMe), 3.59 – 3.50 (m, 1H, H^B), 3.49 – 3.41 (m, 1H, H^B), 3.28-3.22 (m, 1H, H^F), 3.18-3.13 (m, 1H, H^F), 1.80–1.71(m, 3H,$2H^E$+H^C), 1.65 – 1.55 (m, 1H, H^C). ^{13}C NMR (126 MHz, CD_3OD) δ 170.2, 160.0, 147.4, 146.7, 137.7, 134.2, 131.1, 129.3, 128.7, 128.6, 127.6, 114.0, 87.3, 67.8, 61.5, 55.7, 46.3, 38.6, 38.0, 37.9 ppm. HRMS calcd. for $C_{13}H_{20}N_2O_3$ $[M-H]^-$ 553. 2781, found 553.2720.

Synthesis of Linkers A and B

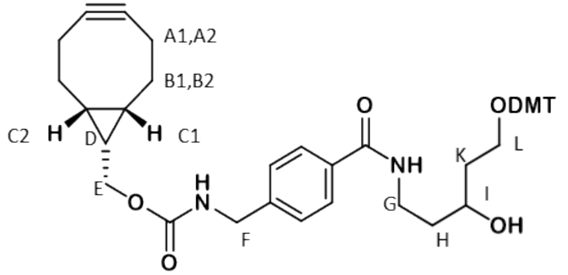

endo-Bicyclo[6.1.0]non-4-yn-9-ylmethyl-(2-(R)-1-O-(4,4'-Dimethoxytrityl)-5N-[(methyl)-benzoyl]-aminopentane-1,3-diol)carbamate (3). BCN carbinol (**2**) ((0.86 g, 5.7 mmol) was activated according to a published procedure [28]. Then 0.5 g of crude activated BCN carbinol (**2**) was dissolved in DCM (25 mL) under N_2 atmosphere. Compound **1** (1.10 g; 1.98 mmol) and TEA (0.70 mL, 5.2 mmol) were added, and the resulting mixture was stirred for 20 min. The volatiles were then evaporated under reduced pressure and the residue was purified by RP column chromatography using 0 to 100% gradient of MeCN (containing 0.1% TEA) in water (containing 0.1% TEA) to yield compound **3** as a white foam. Yield over two steps: 1 g (24%). ^{1}H NMR (500 MHz, CD_3OD) δ 7.82 – 7.70 (m, 2H), 7.43 – 7.32 (m, 4H), 7.30 – 7.12 (m, 7H), 6.81-6.77 (m, 4H), 4.32 (s, 2H, H^{F}), 3.98 (d, *J* = 7.1 Hz, 2H, H^{E}), 3.94-3.88 (m, 1H, H^{I}), 3.74 (s, 6H, -OMe), 3.57-3.48 (m, $1H^{G}$), 3.48-3.40 (m, $1H^{G}$), 3.28-3.21 (m, 1H, H^{L}), 3.18-3.11 (m, 1H, H^{L}), 2.34 (d, *J* = 13.6 Hz, 2H, $H^{B1'', B2''}$), 2.27-2.02 (m, 4H, $H^{A1,A2}$), 1.81-1.69 (m, 3H, $2H^{K}$+H^{H}), 1.66 – 1.55 (m, 1H, H^{H}), 1.40-1.28 (m, 2H, $H^{B1', B2'}$), 0.78-0.64 (m, 3H, $H^{C1,C2,D}$).^{13}C NMR (126 MHz, CD_3OD) δ 170.1, 160.0, 159.4, 146.8, 144.7, 137.7, 137.7, 134.4, 131.2, 129.3, 128.7, 128.5, 128.3, 127.7, 114.0, 99.4, 87.3, 70.2, 67.9, 61.5, 55.7, 45.0, 38.6, 38.0, 37.9, 34.4, 25.1, 24.2, 21.9 ppm. HRMS calcd. for $C_{45}H_{50}N_2O_7$ $[M+H]^+$ 731.3696, found 731.3668.

endo-Bicyclo[6.1.0]non-4-yn-9-ylmethyl-(2-(R)-1-O-(4,4'-Dimethoxytrityl)-5N-[(methyl)-benzoyl]-aminopentane-1,3-diol)amidite (linker A): Compound **3** (1.00 g, 1.36 mmol) was dissolved in DCM (10 mL) and cooled in an ice bath (0 °C). Diisopropyl ethyl amine (DIPEA, 1.2 mL, 6.9 mmol) was added to the resulting solution under N_2 atmosphere followed by the addition of 2-cyanoethyl N,N-diisopropylphosphoramidochloridite (610 µL, 2.74 mmol). The reaction mixture was stirred for 3 h and quenched with methanol (55 µL). Then the reaction mixture washed with aq. 10% $NaHCO_3$ solution and chromatographed (ISCO chromatography system) using a gradient of 0-70% ethyl acetate in DCM to give linker A. (0.46 g, 36%) as a white foam. ^{31}P NMR (202 MHz, CD_3CN) δ 147.8, 146.7 ppm. HRMS calcd. for $C_{54}H_{67}N_4O_8P$ $[M+H]^+$ 931.4775, found 931.4775.

4,5-Dibromobicyclo[6.1.0]nonane-9-carboxylic acid (5): Bicyclo[6.1.0]non-4-ene-9-carboxylic acid **4** (5.0 g, 30 mmol) was dissolved in 50 mL DCM and cooled to 0 °C. 1.7 mL (33.09 mmol) of Br_2 was added dropwise under N_2 atmosphere. Then the ice bath was removed, and the reaction mixture was left to stir at ambient temperature for 1 h following by quenching with 50 mL of 10 % aq. $Na_2S_2O_3$ solution. Then the phases were separated, and water phase was extracted with DCM twice. The organic phases were combined, dried over $MgSO_4$ and filtered. Volatiles evaporated under reduced pressure to give 7.4 g of crude material as a white powder which was used for the next step without further purification.

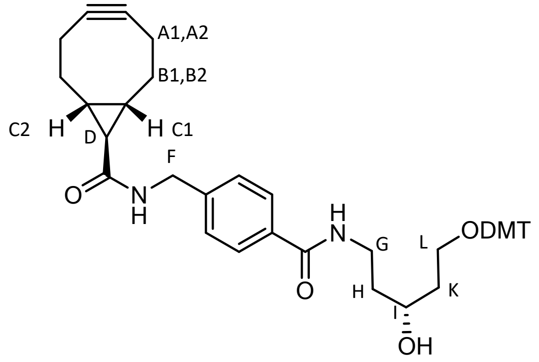

***exo*-Bicyclo[6.1.0]non-4-yn-9-ylmethyl-(2-(R)-1-O-(4,4'-Dimethoxytrityl)-5N-[(methyl)-benzoyl]-aminopentane-1,3-diol (7):** Bicyclo[6.1.0]non-4-yne-9-carboxylic acid **6** was prepared using a published procedure [29]. Then BCN acid **6** (0.45 g, 7.7mmol) was dissolved in N-methylpyrrolidone (NMP, 3 mL) followed by the addition of 1-Ethyl-3-(3-dimethylaminopropyl)carbodiimide (EDCI, 0.51 g, 3.3 mmol) and hydroxybenzotriazole (HOBt, 0.74 g, 0.55 mmol). The reaction mixture remained heterogeneous, therefore, additional NMP (7 mL) was added. Aminodiol linker **1** (1.67 g, 3.02 mmol) was dissolved in NMP (9 mL), slowly added to the reaction mixture, and left to stir overnight. The resulting homogenous solution was diluted with DCM (approximately 30 mL) and washed with water (x4 times, around 25 mL each time) and then once with brine. Then the organic phase was concentrated under reduced pressure and applied to RP column. Purified by using 0-100 % gradient of MeCN in water with 1%TEA. Fractions containing the product were combined and freeze dried to give a white cotton-like material **7** (0.76 g, 40%). ^{1}H NMR (500 MHz, DMSO-d_6) δ 8.49 (t, J = 6.0 Hz, 1H, NH), 8.37 (t, J = 5.6 Hz, 1H, NH), 7.84 – 7.72 (m, 2H), 7.41 – 7.33 (m, 2H), 7.33 – 7.25 (m, 4H), 7.25 – 7.17 (m, 5H), 6.91 – 6.80 (m, 4H), 4.45 (d, J = 5.6 Hz, 1H, OH), 4.30 (d, J = 6.0 Hz, 2H, H^F), 3.72 (s, 6H, -OMe), 3.69 – 3.64 (m, 1H, H^I), 3.41 – 3.26 (m, 2H+H_2O, H^G), 3.05 (t, J = 7.0 Hz, 2H,H^L), 2.36 – 2.21 (m, 4H, $H^{A1,A2}$), 2.10 (d, J = 15.8 Hz, 2H, $H^{B1'',B2''}$), 1.74 – 1.57 (m, 3H, 2H^K+H^H), 1.56 – 1.44 (m, 1H, H^H), 1.43 – 1.32 (m, 2H, $H^{B1',B2'}$), 1.25 – 1.16 (m, 3H, $H^{C1,C2,D}$). ^{13}C NMR (126 MHz, $CDCl_3$) δ 173.1, 167.3, 158.6, 144.7, 142.0, 136.1, 133.8, 130.0, 128.0, 127.4, 127.0, 113.3, 98.7, 86.9, 70.8, 62.4, 55.3, 43.6, 38.0, 36.7, 36.0, 32.7, 28.7, 26.6, 21.2 ppm. HRMS calculated for $C_{44}H_{48}N_2O_6$ $[M+H]^+$ 701.3591, found 701.3586.

***exo*-Bicyclo[6.1.0]non-4-yn-9-ylmethyl-(2-(R)-1-O-(4,4'-Dimethoxytrityl)-5N-[(methyl)-benzoyl]-aminopentane-1,3-diol amidite (linker B):** To a solution of BCN derivative **7** (0.67 g, 0.66 mmol) in 7 mL DCM, 0.67 mL of 1.0 M 4,5-dicyanoimidazole (DCI) solution in MeCN was added followed by a dropwise addition of 0.46 mL (1.434 mmol) bis(diisopropylamino)(2-cyanoethoxy)phosphine. Then the reaction mixture was stirred at ambient temperature for 1.5 h and quenched with TEA (0.93 mL of) following with washing with 10% aq. $NaHCO_3$ (x2) and water (x1). Obtained organic phase dried over $MgSO_4$, filtered, and concentrated under reduced pressure. The resulting crude material was chromatographed on RP column using 0-100 % gradient MeCN in water with 1%TEA. Fractions containing the product were combined and concentrated under reduced pressure to give linker B as white solids (0.28 g, 32%). ^{31}P NMR (202 MHz, CD_3CN) δ=147.8, 146.6 ppm. HRMS calculated for $C_{53}H_{65}N_4O_7P$ $[M+H]^+$ 901.4669, found 901.4693.

S-2. NMR spectra of linkers and intermediates

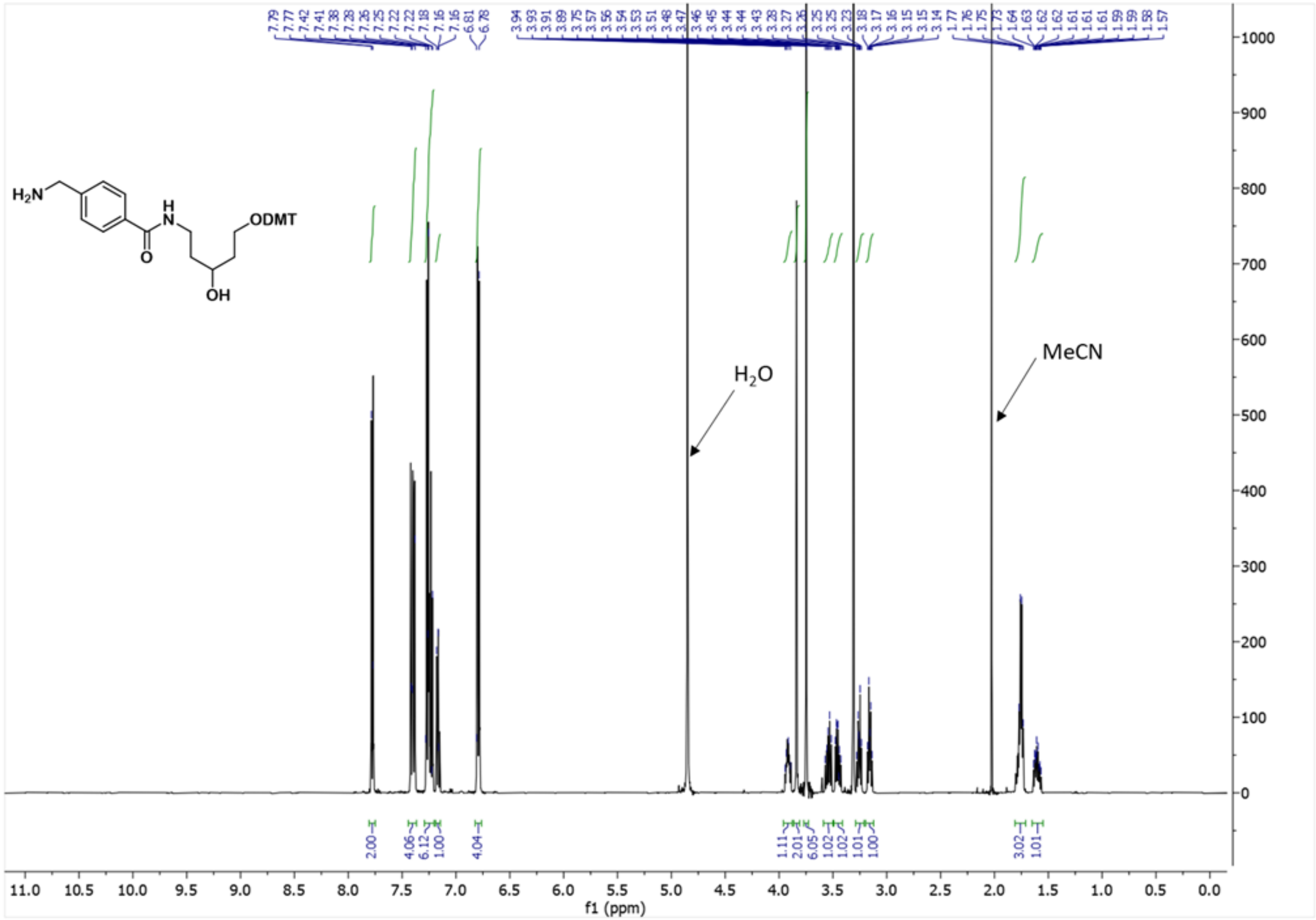

Figure S2. ^{1}H NMR (500.1 MHz, CD_3OD) spectrum of common intermediate 1.

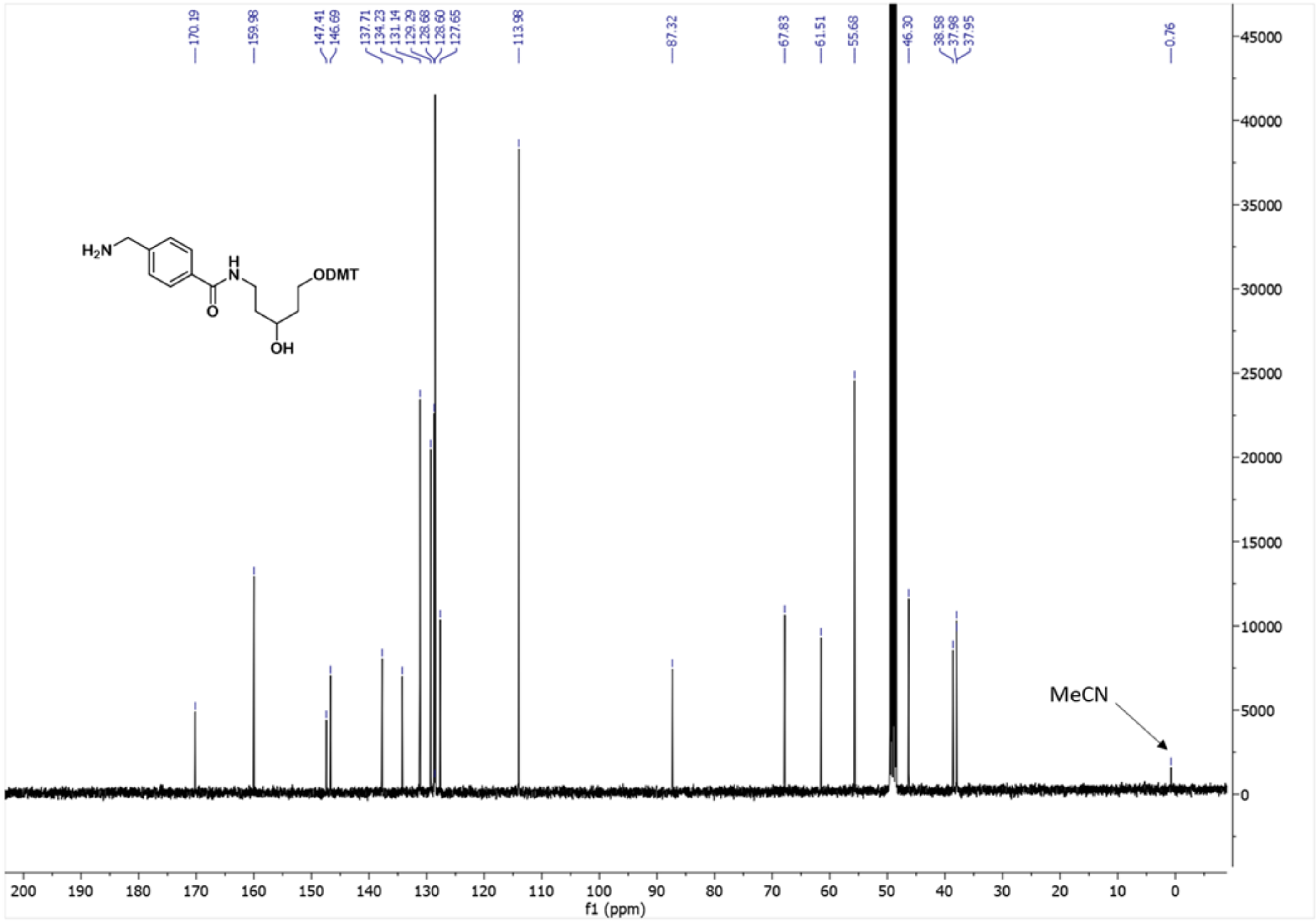

Figure S3. ^{13}C NMR (126 MHz, CD_3OD) spectrum of common intermediate 1.

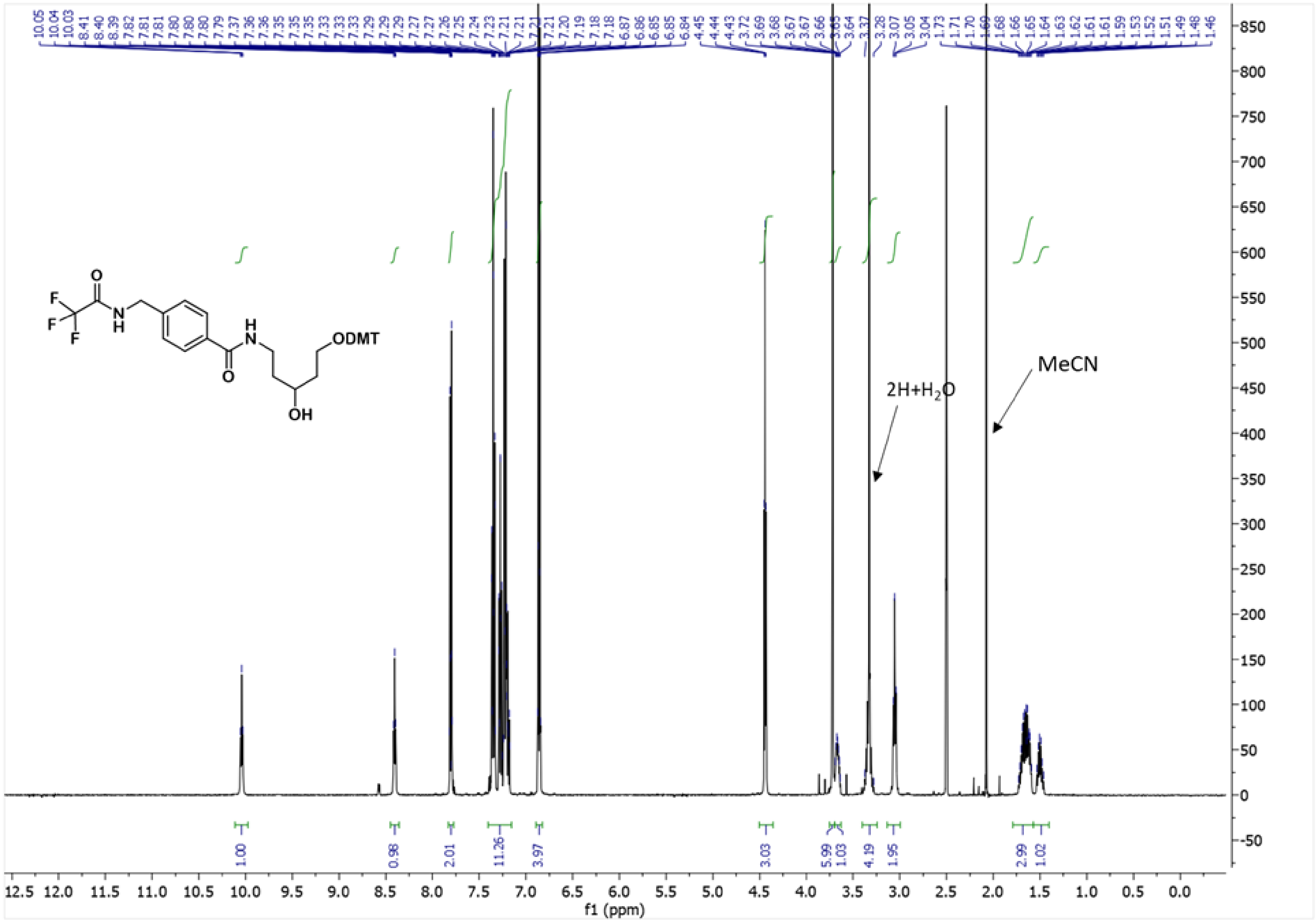

Figure S4. ^{1}H NMR (500.1 MHz, DMSO-d_6) spectrum of compound 12.

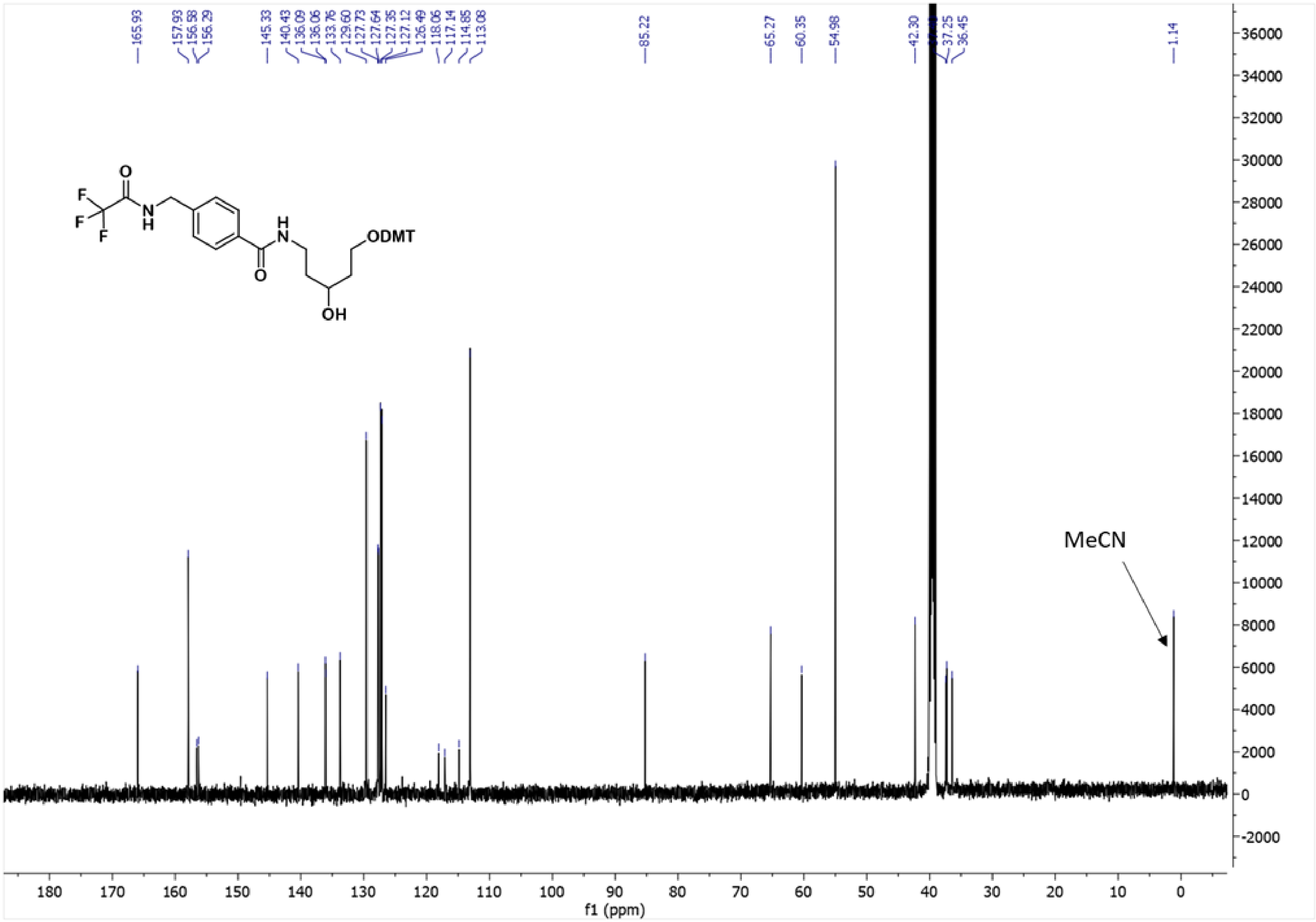

Figure S5. ^{13}C NMR (126 MHz, DMSO-d_6) spectrum of compound 12.

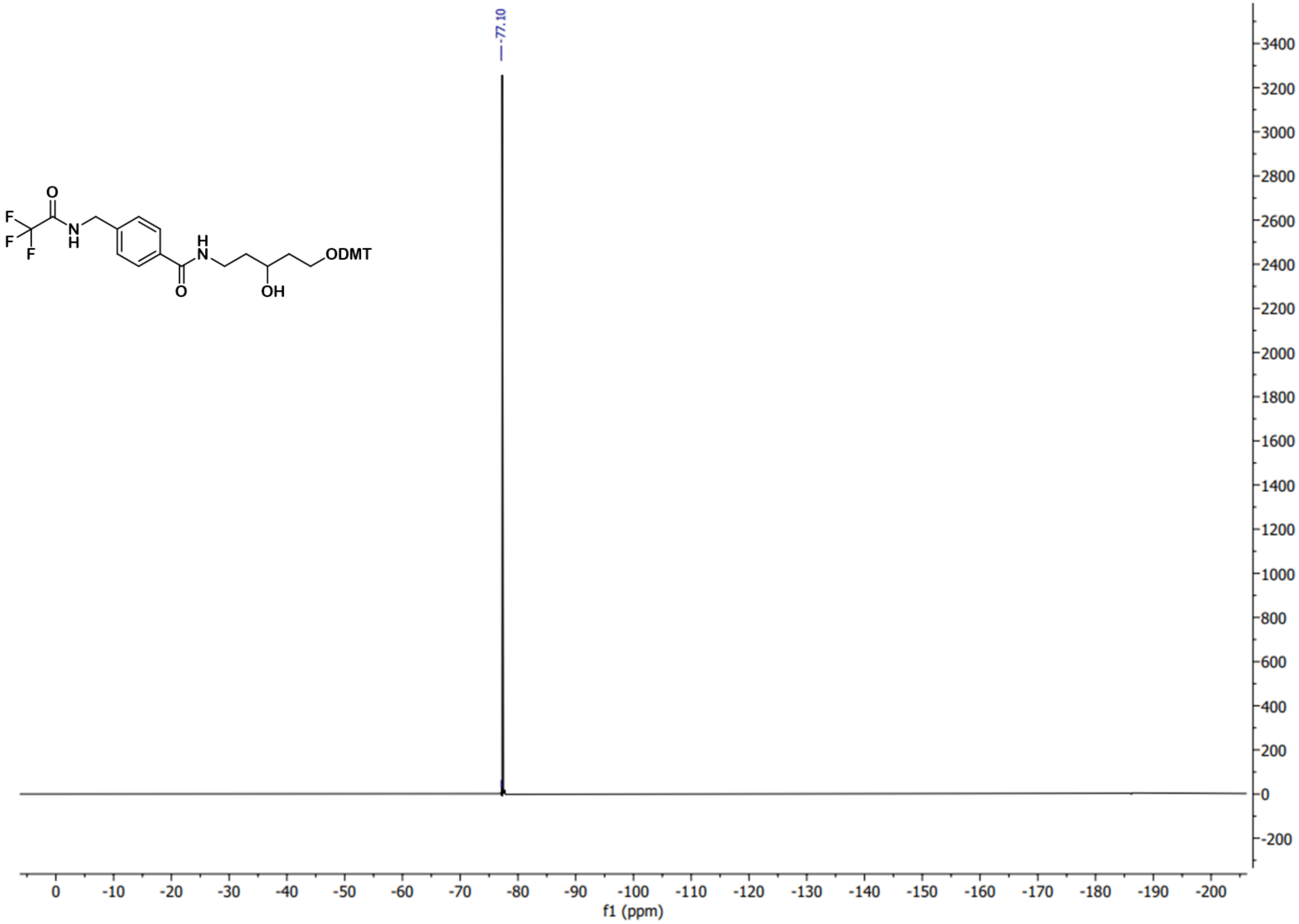

Figure S6. ^{19}F NMR (470.56 MHz, CD_3OD) of compound 12.

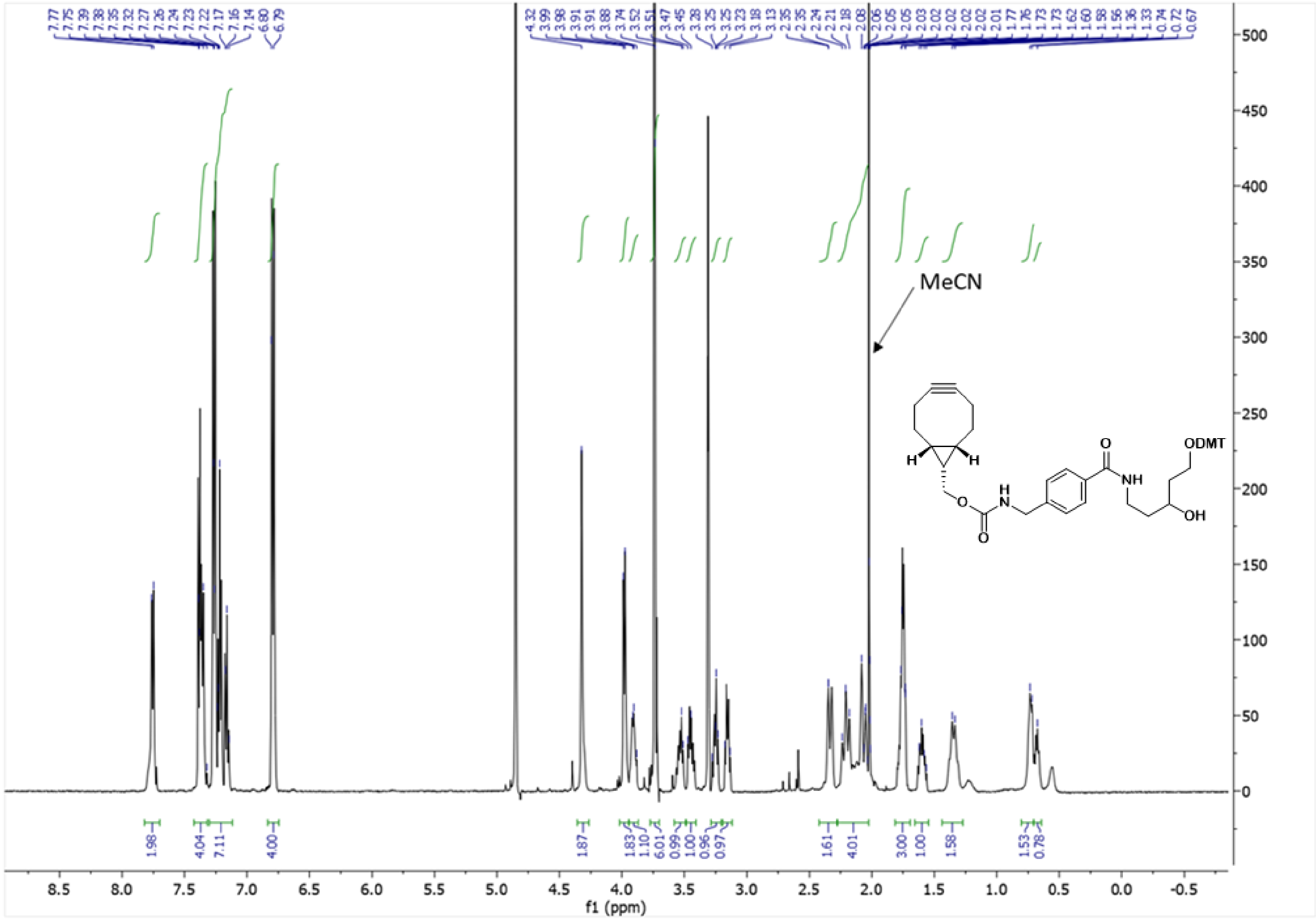

Figure S7. ^{1}H NMR (500 MHz, CD_3OD) of compound 3.

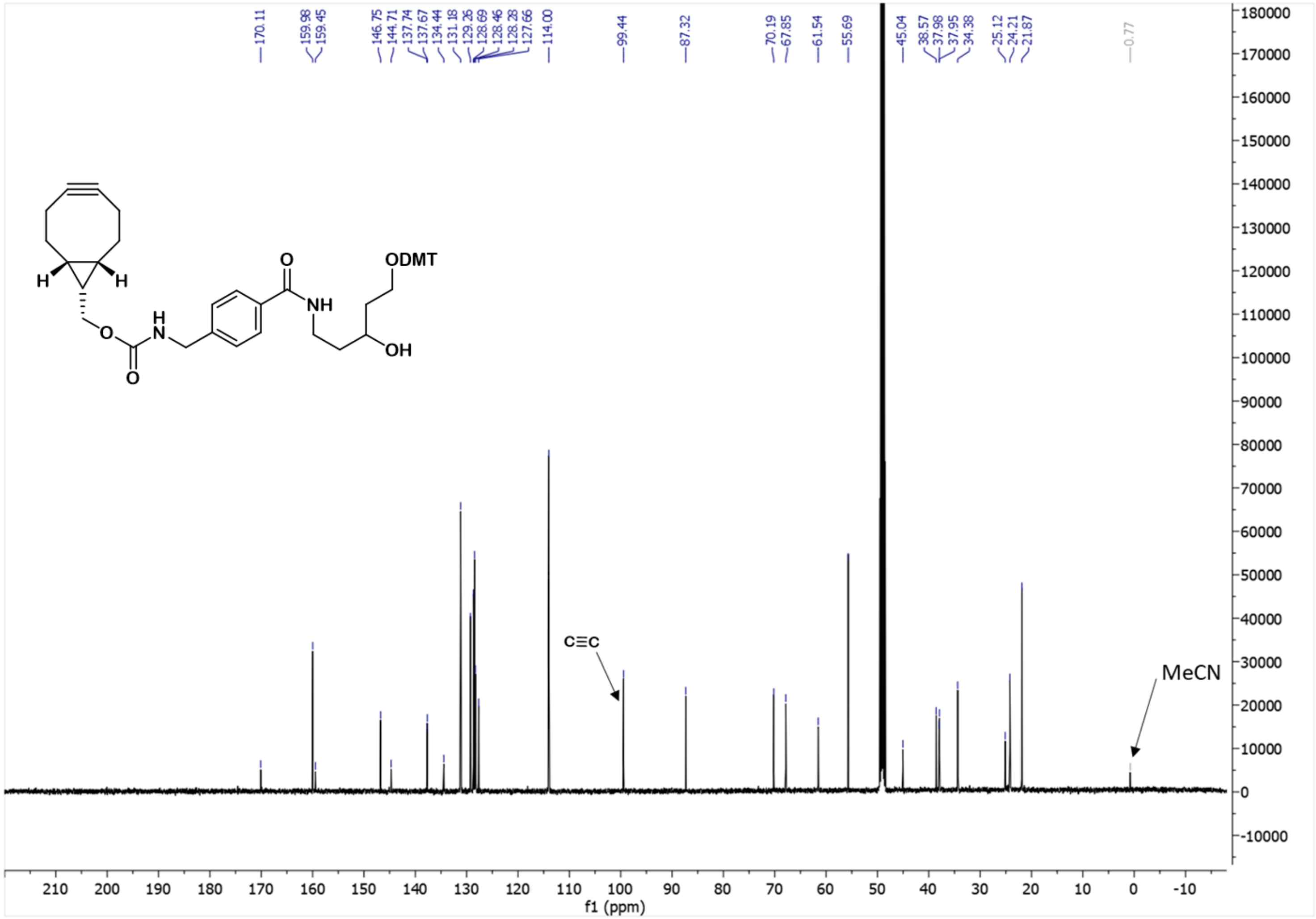

Figure S8. ^{13}C NMR (126 MHz, CD_3OD) of compound 3.

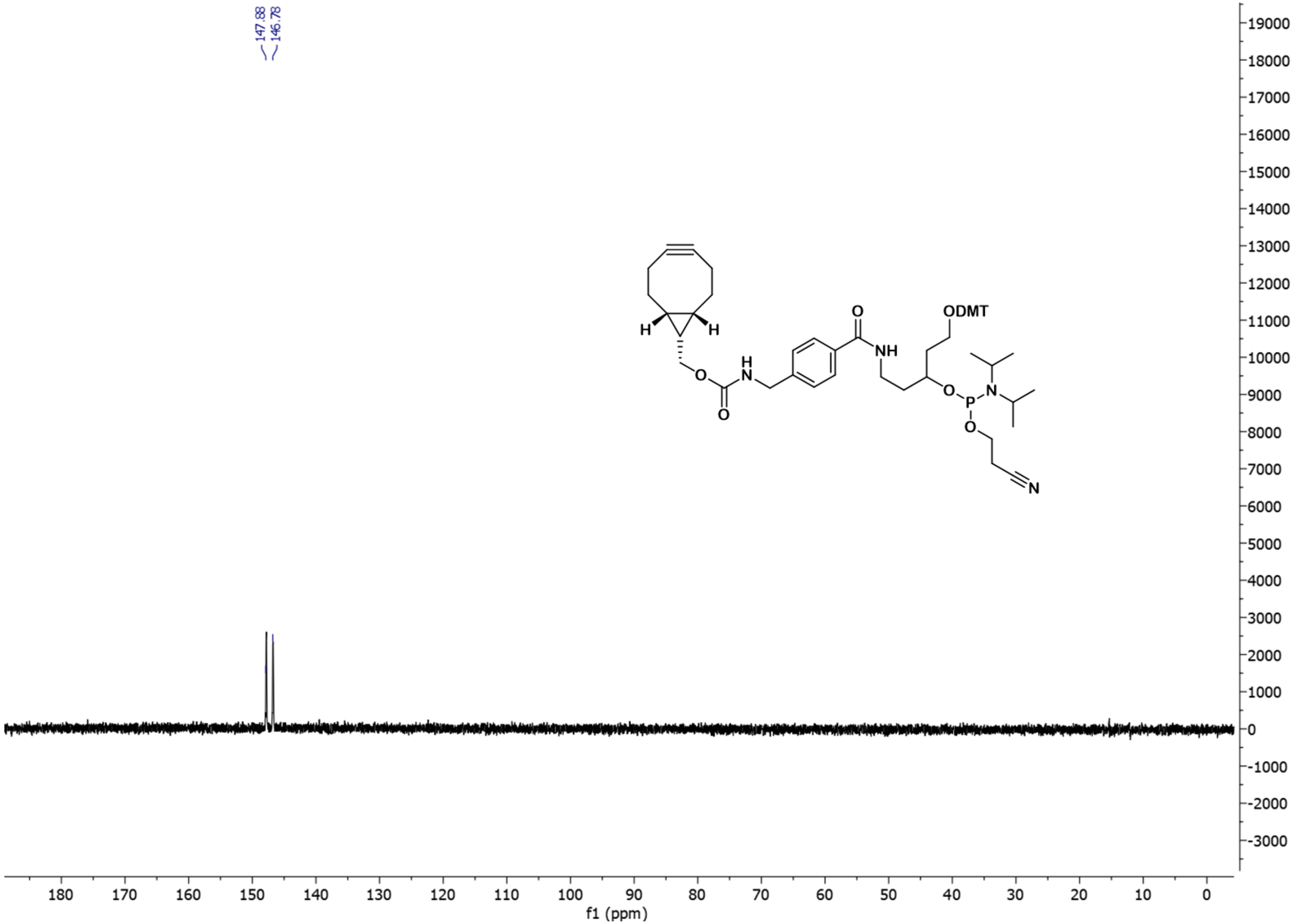

Figure S9. ^{31}P NMR (202 MHz, CD_3CN) of Linker A.

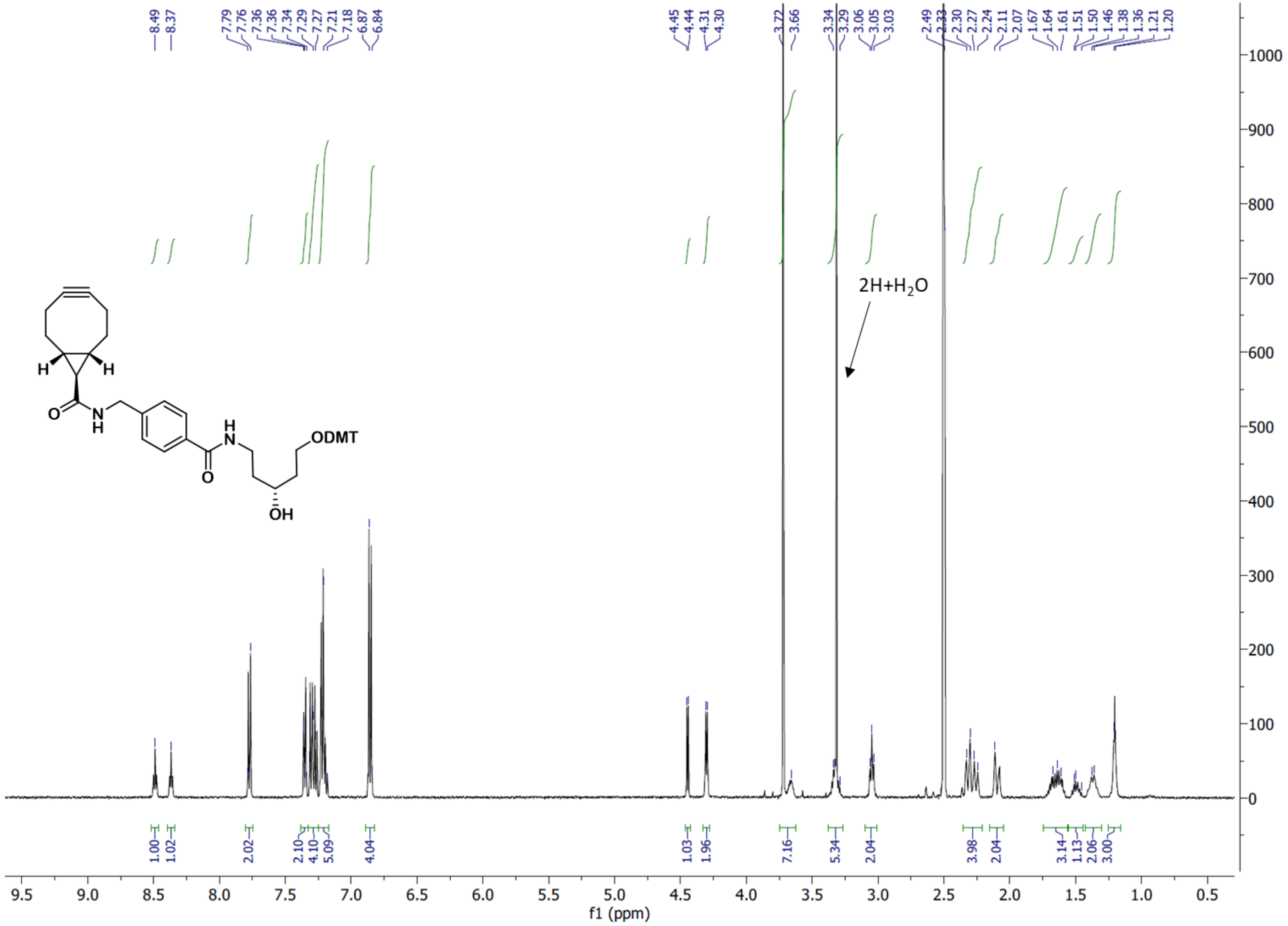

Figure S10. ^{1}H NMR (500 MHz, DMSO- d_6) of compound 7.

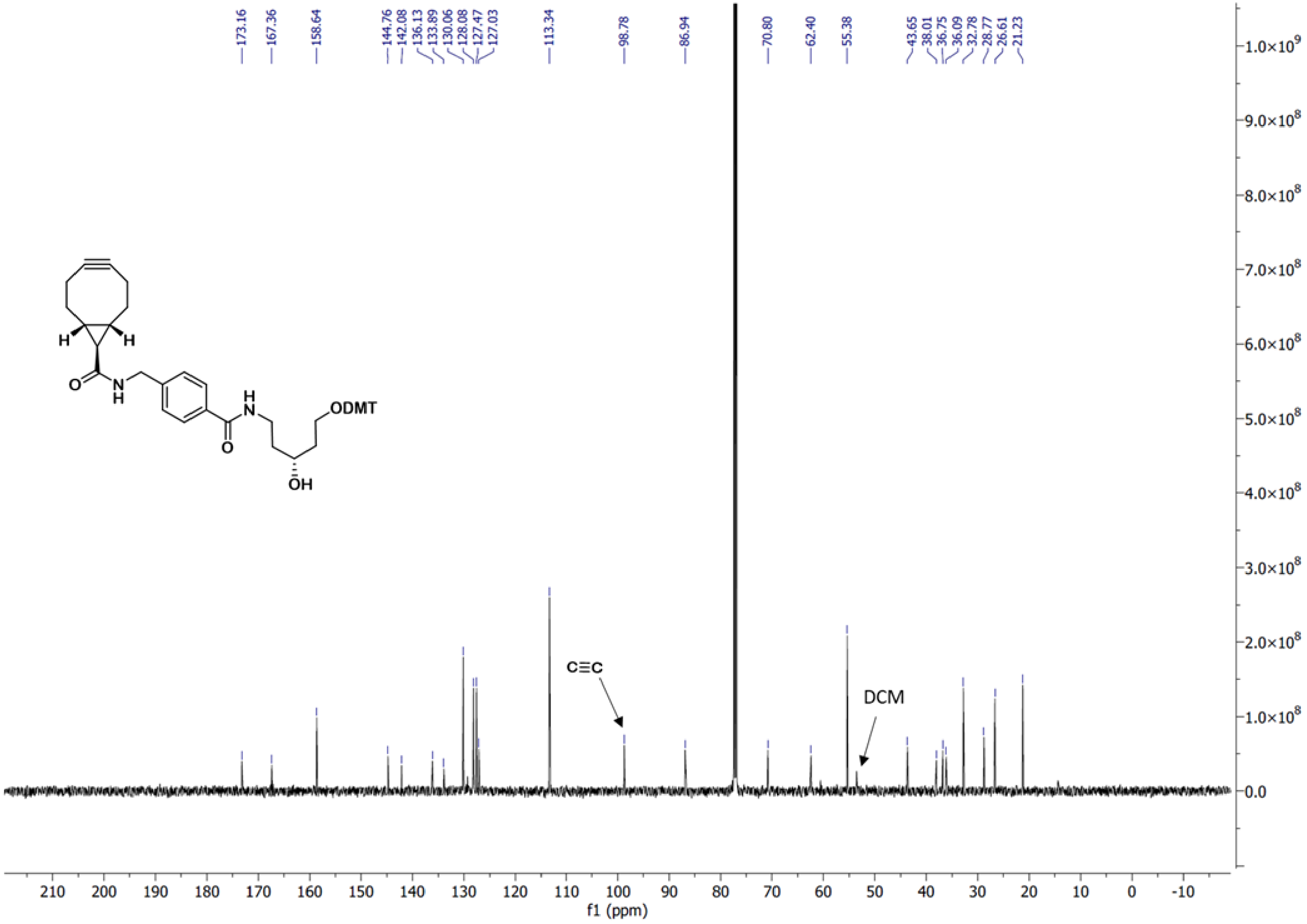

Figure S11. ^{13}C NMR (126 MHz, $CDCl_3$) of compound 7.

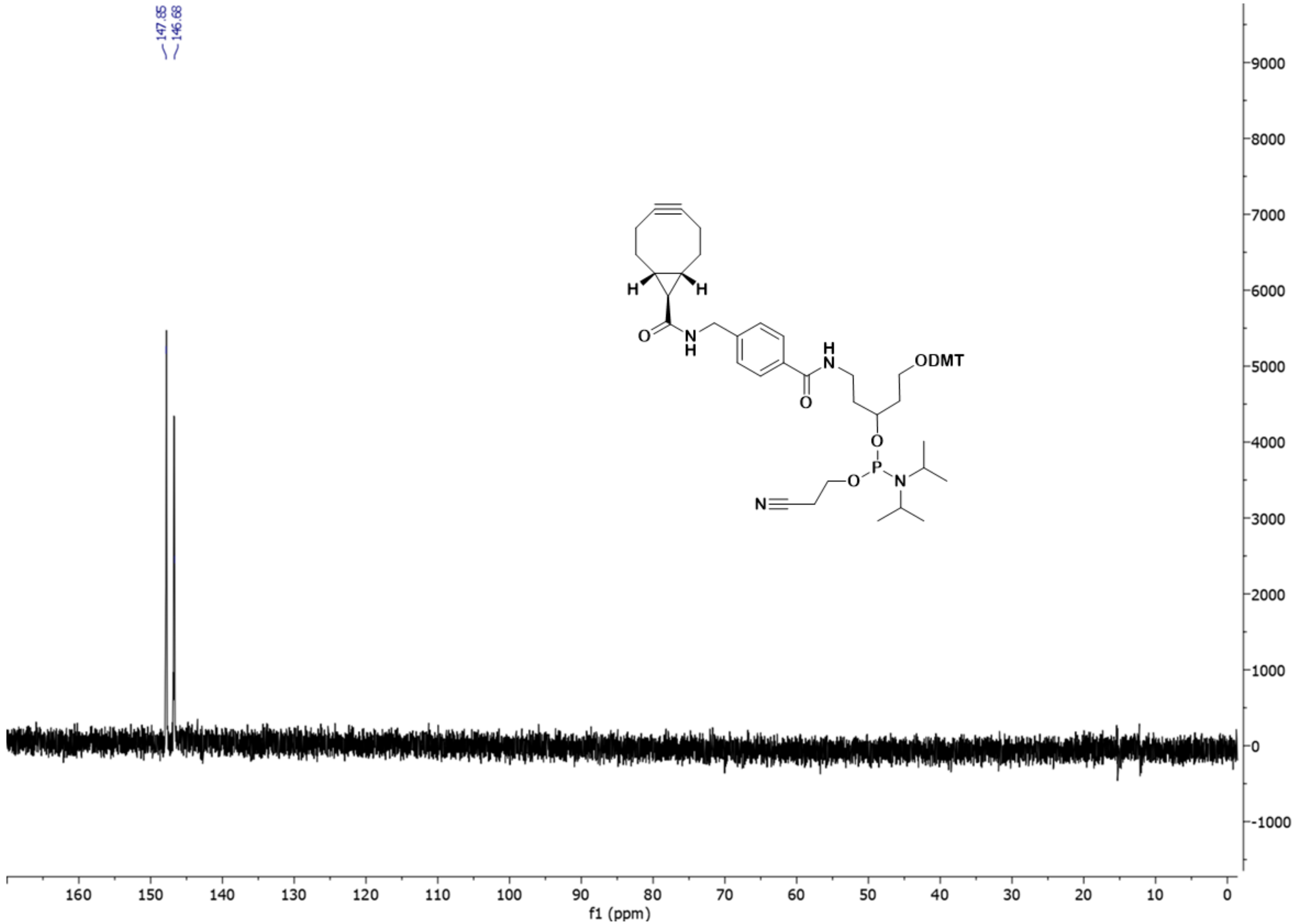

Figure S12. ^{31}P NMR (202 MHz, CD_3CN) of Linker B.

S-3. Kinetic studies of BCN carbinol

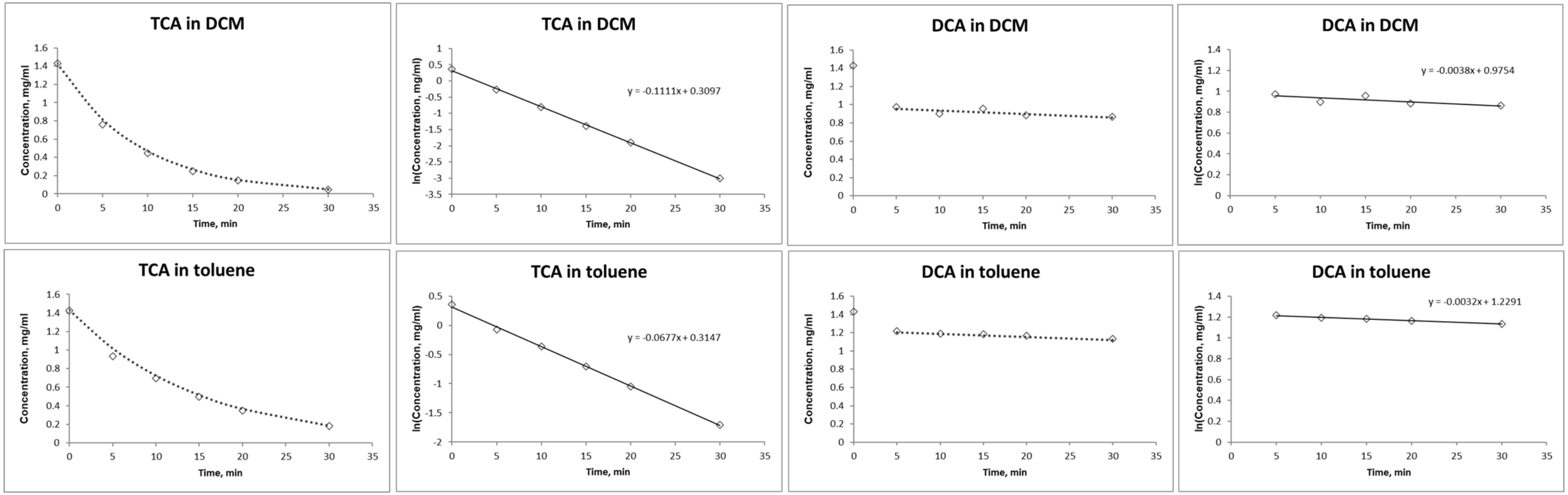

Figure S13. Kinetic profiles and kinetic constants of BCN-OH stability studies in the presence of acidic solutions.

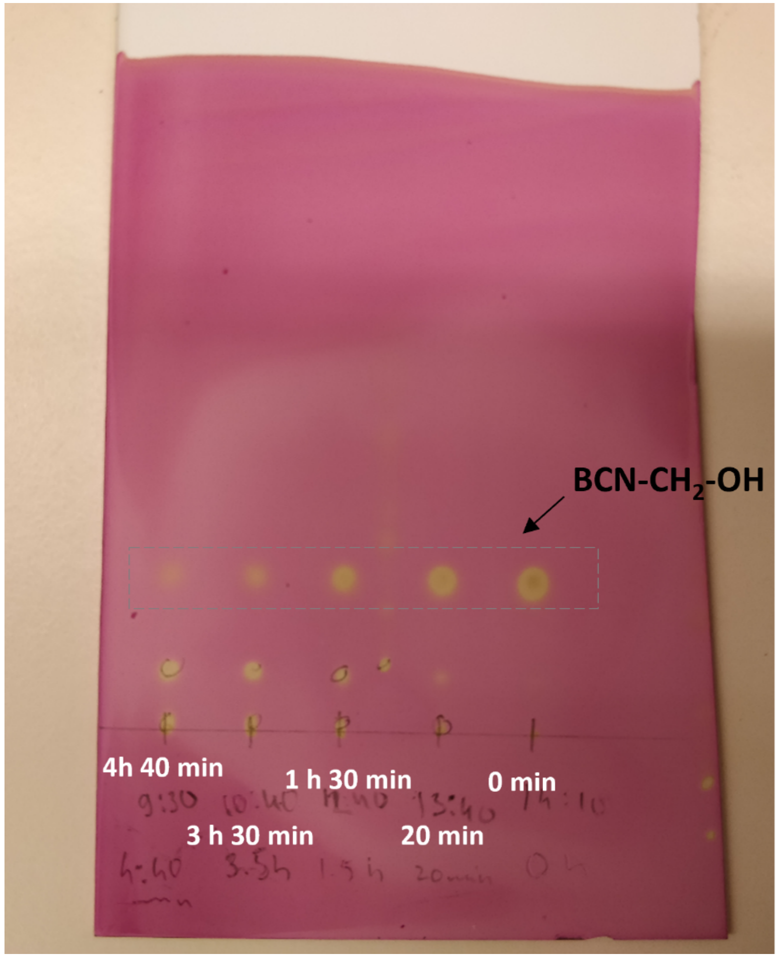

Figure S14. Stability of BCN carbinol on TLC plate over time. Eluent: EtOAc/heptane (1:1, v/v).

S-4. RP-HPLC profiles and MS analyses of oligomer stability studies

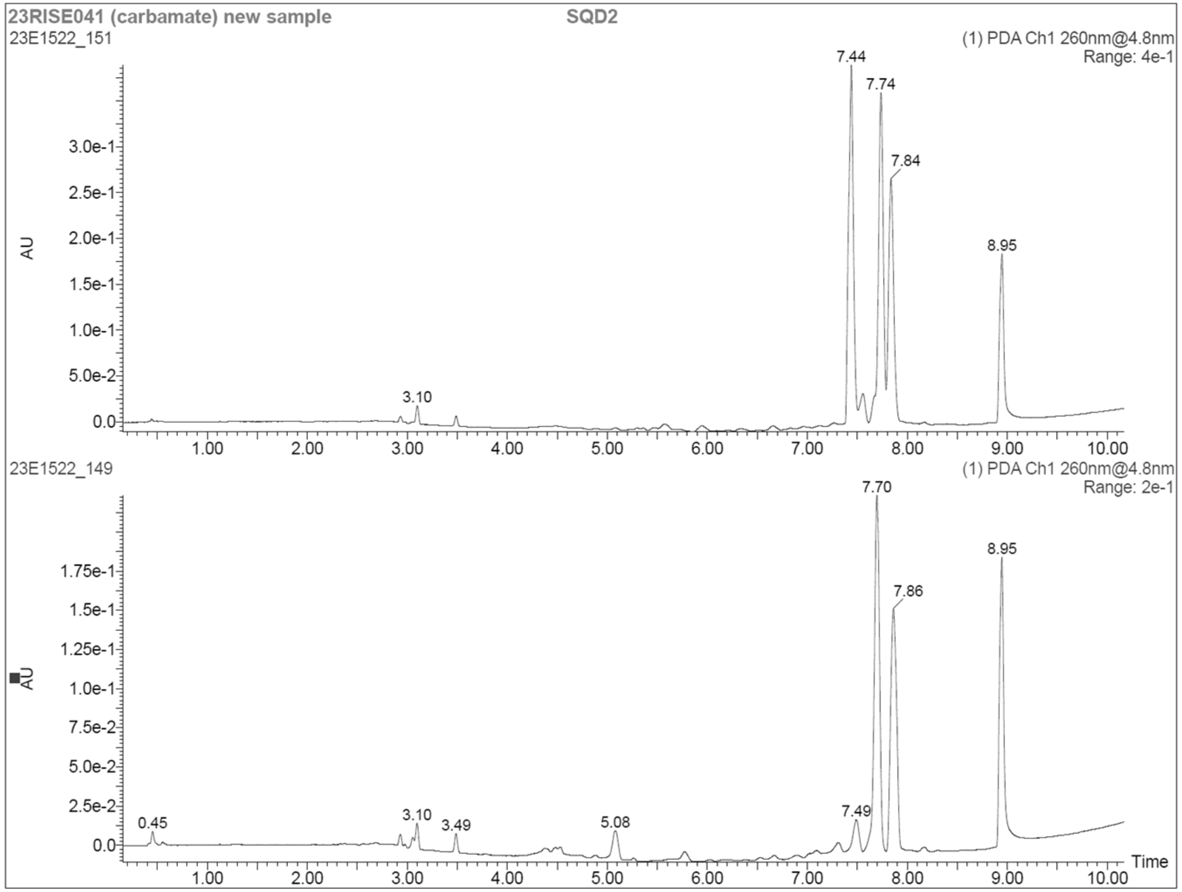

Figure S15. Comparison of HPLC profiles of $(A)_2$-T_5 before and after SPAAC. Trityl (DMT) is intact in both scenarios. Above: HPLC profile of $(A)_2$-T_5 + Fmoc-L-Lys(N_3)-OH (DMT-on). 7.44 min: Fmoc-L-Lys(N_3)-OH, 7.74 and 7.84min: bis-conjugate, 8.95 min: unidentified. Bellow: HPLC profile of $(A)_2$-T_5 (DMT-on): 7.7 and 7.86 min: $(A)_2$-T_5, 8.95 min: unidentified. 0.4 ml/min gradient from 0 -90-95% MeOH in 8.6 mM TEA + 100 mM HFIP buffer over 15 min at 40 °C.

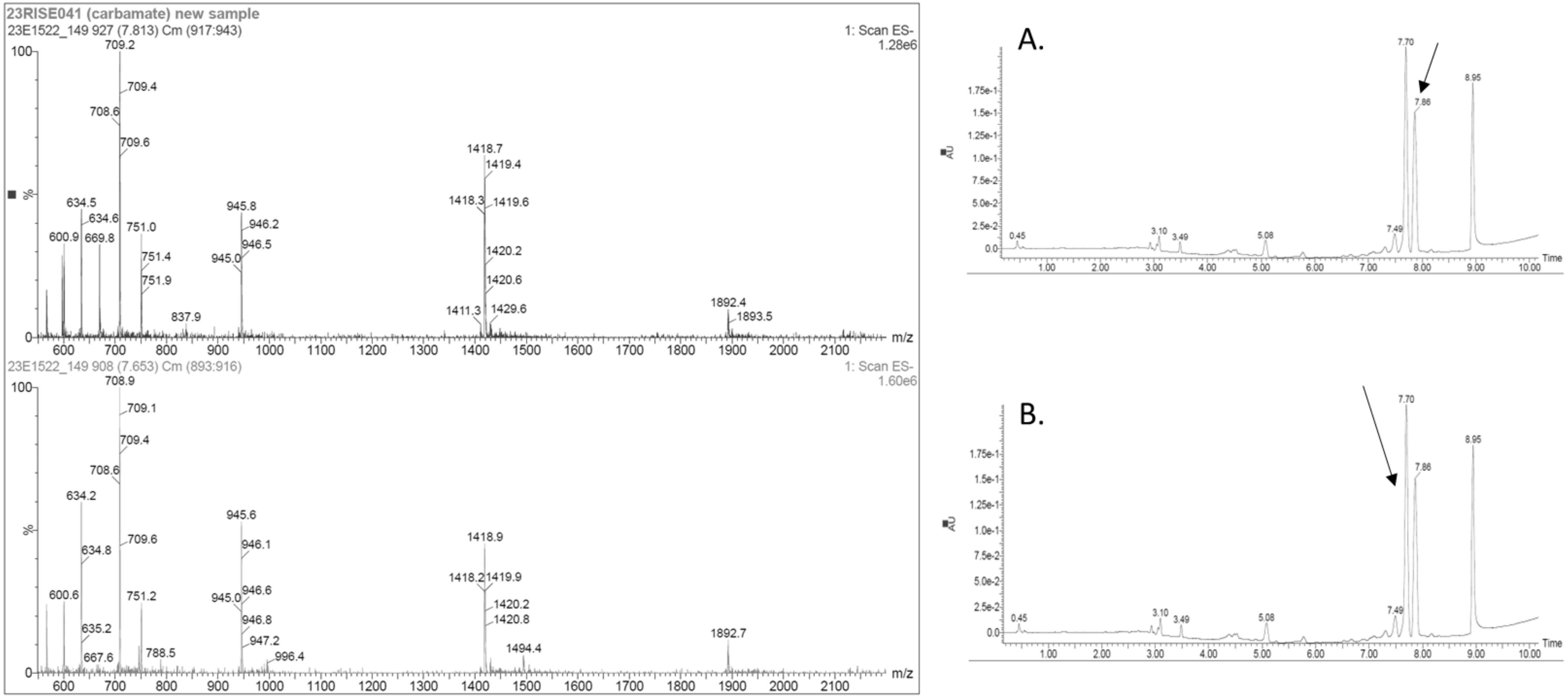

Figure S16. MS analysis and RP-HPLC profile of (A)$_2$-T$_5$ (DMT on, both diasteromers,(diastereomers are separating on HPLC because of phosphorothioate linkages). The arrows indicate on which isomer MS profile is shown. Mw[M-2H]$^{2-}$ calc. 1418.4, obs. 1418.7 and 1418.9.

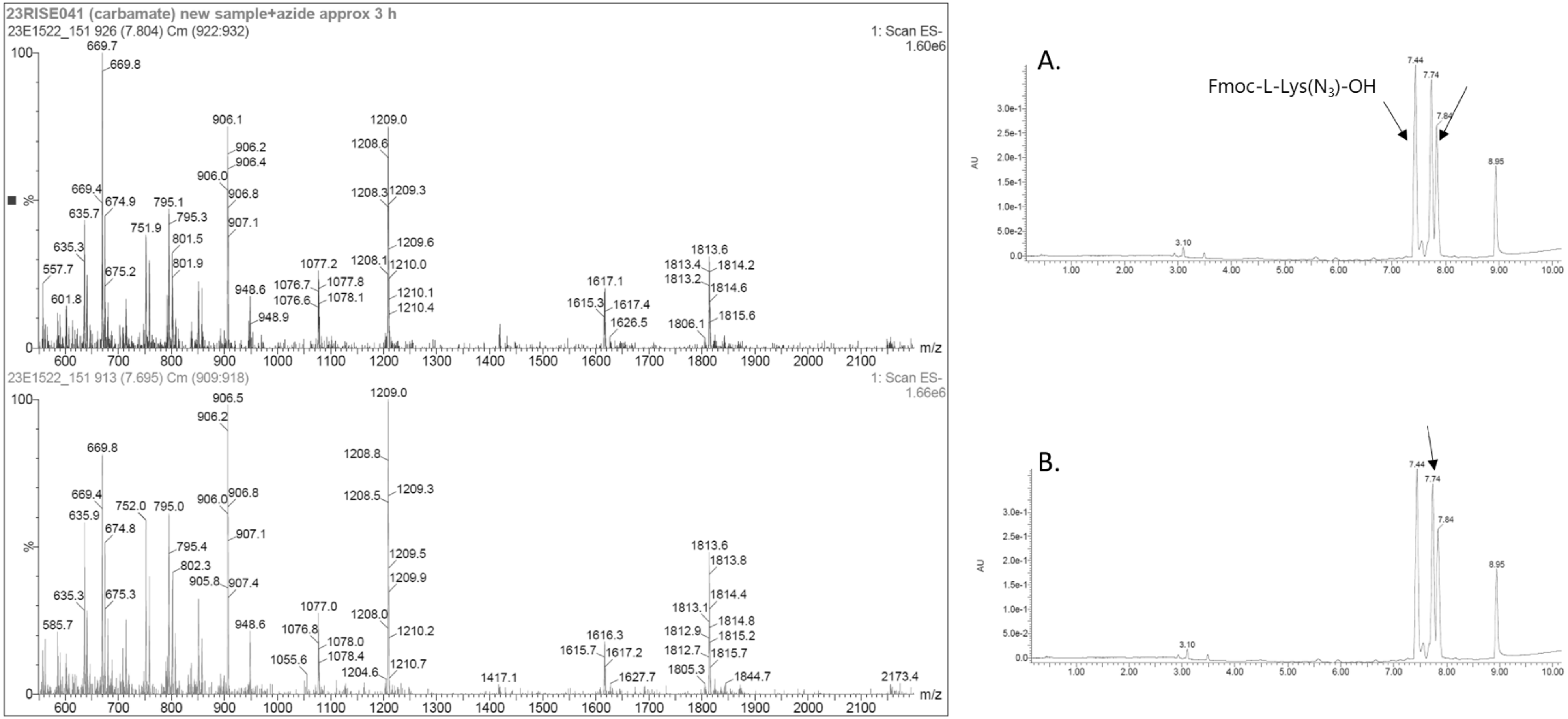

Figure S17. MS and RP-HPLC profile of (A)$_2$-T$_5$ bis-conjugate (DMT-on, both diastereomerss) (diastereomers are separating on HPLC because of phosphorothioate linkages). The arrows indicate on which isomer MS profile is shown. Mw[M-3H]$^{3-}$ calc. 1209.0, obs.1209.0 and 1209.0.

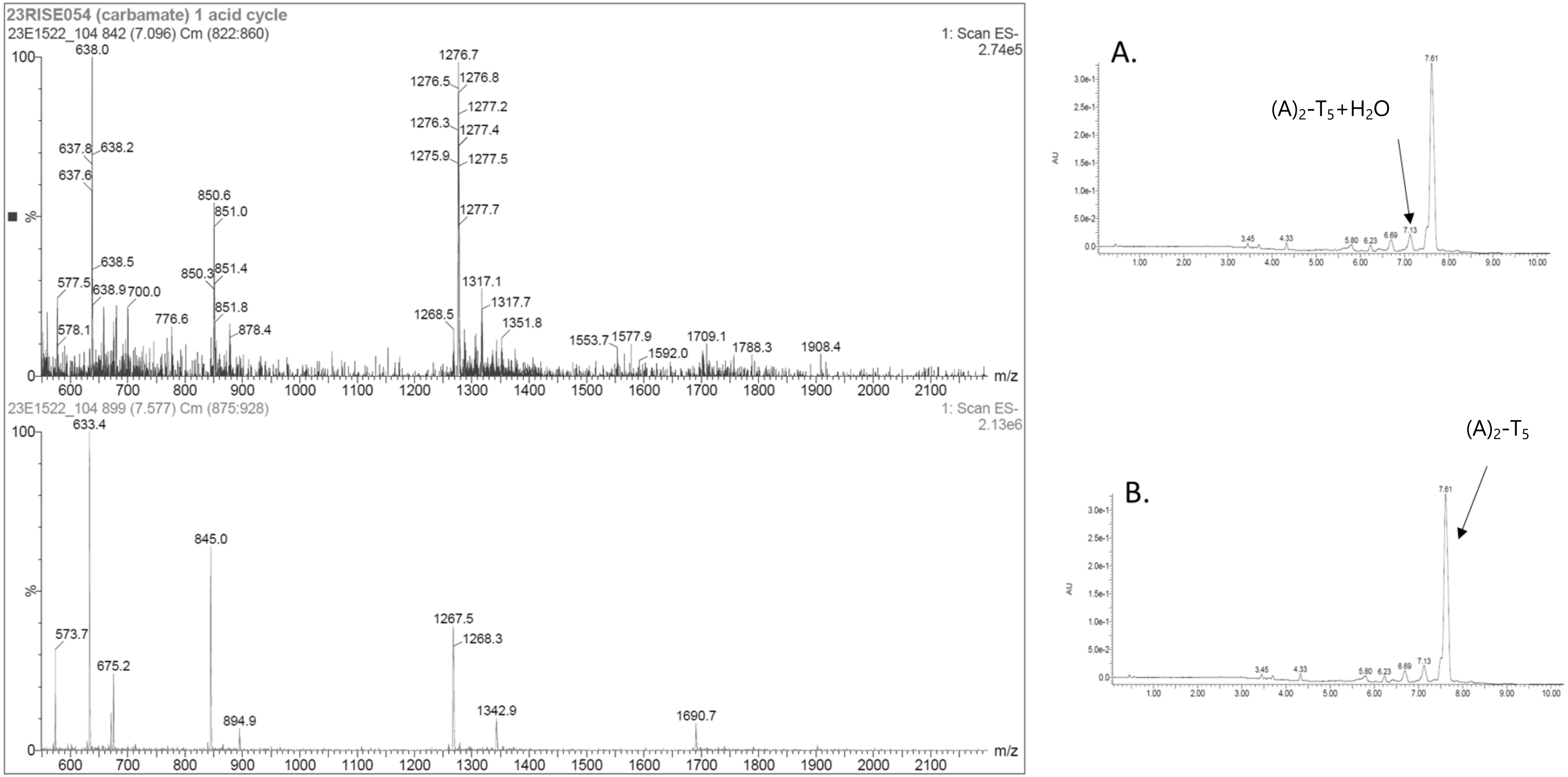

Figure S18. MS and RP-HPLC profile of $(A)_2$-T_5 after one detritylation cycle. The arrows indicate on which peak MS profile is shown. A.: Mw$[M-2H]^{2-}$ calc. 1276.2, obs. 1276.7. B.: Mw$[M-2H]^{2-}$ calc. 1267.2, obs. 1267.5. 0.4 ml/min gradient from 0 -60-80% MeOH in 8.6 mM TEA + 100 mM HFIP buffer over 15 min at 40 °C.

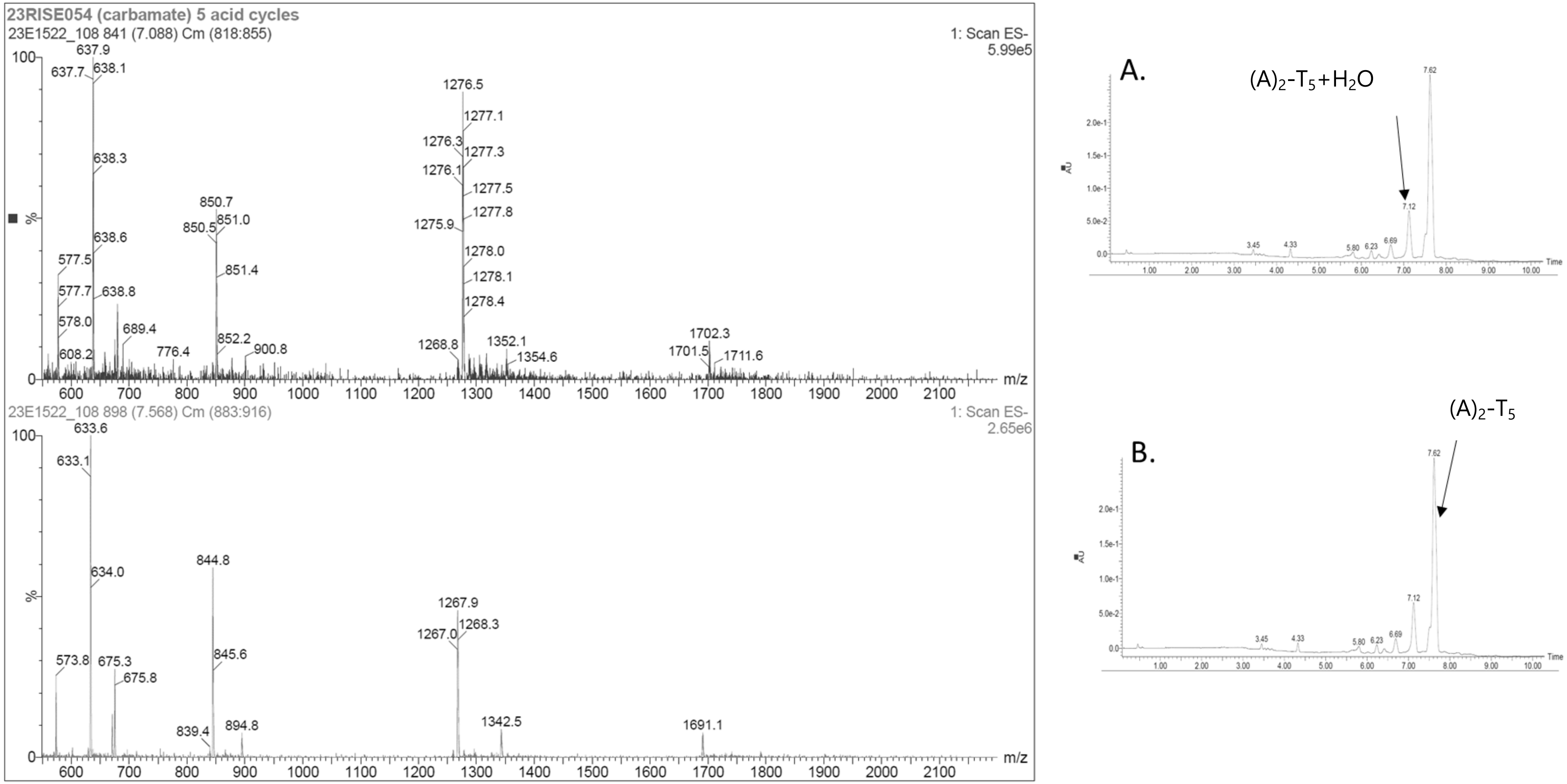

Figure S19. MS and RP-HPLC profile of (A)$_2$-T$_5$ after five detritylation cycles. The arrows indicate on which peak MS profile is shown. A.: Mw[M-2H]$^{2-}$ calc. 1276.2, obs. 1276.5. B.: Mw[M-2H]$^{2-}$ calc. 1267.2, obs. 1267.9. 0.4 ml/min gradient from 0 -60-80% MeOH in 8.6 mM TEA + 100 mM HFIP buffer over 15 min at 40 °C.

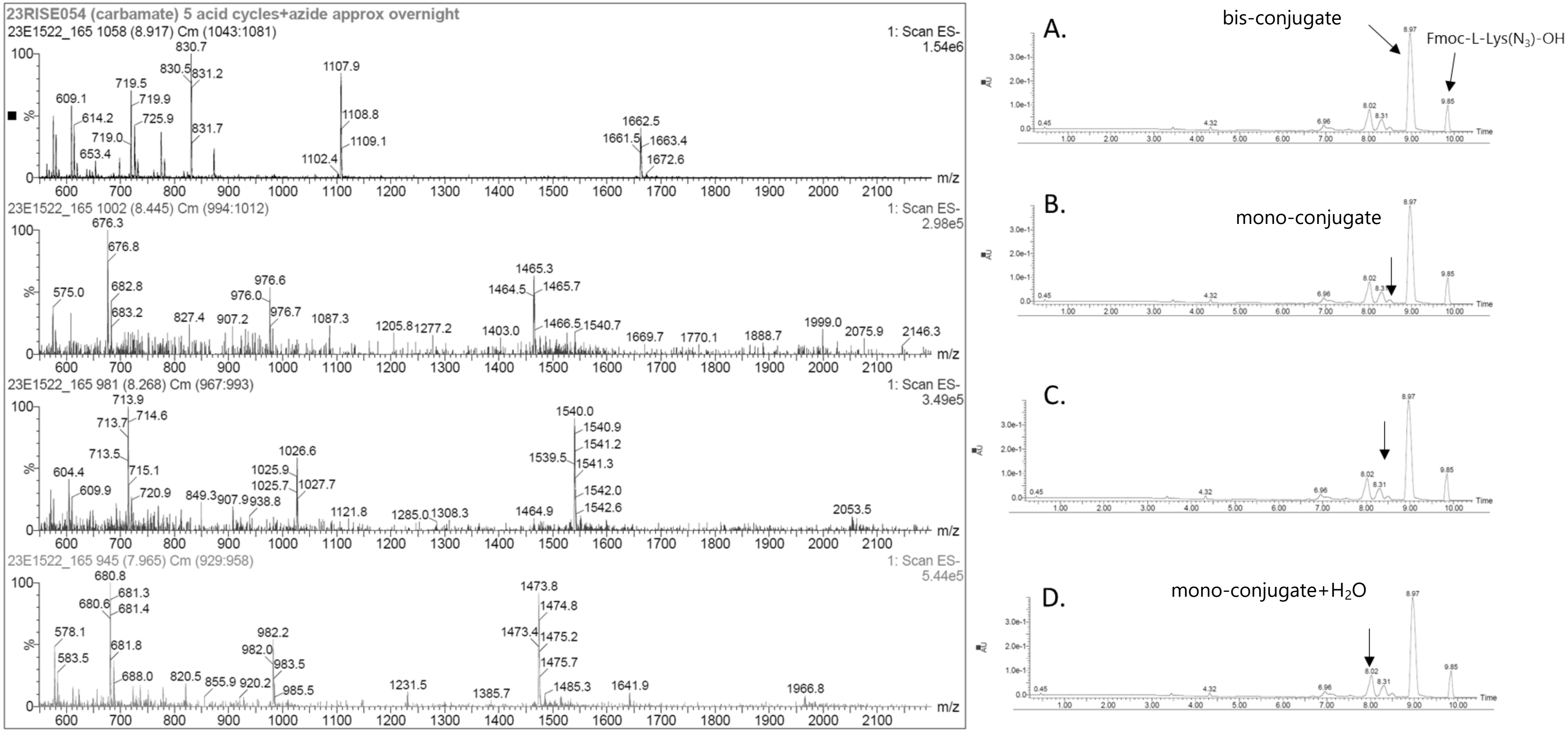

Figure S20. MS and RP-HPLC profiles of $(A)_2$-T_5 after five detritylation cycles + Fmoc-L-Lys(N_3)-OH. A.: bis-conjugate, Mw$[M-3H]^{3-}$ calc. 1107.4, obs.1107.9. B.: monoconjugate, Mw$[M-2H]^{2-}$ calc. 1464.4, obs. 1465.3. C. unidentified impurity. D.: monoconjugate+H_2O, Mw$[M-2H]^{2-}$ calc. 1473.4, obs 1473.8. 0.4 ml/min gradient from 0 -60-80% MeOH in 8.6 mM TEA + 100 mM HFIP buffer over 15 min at 40 °C.

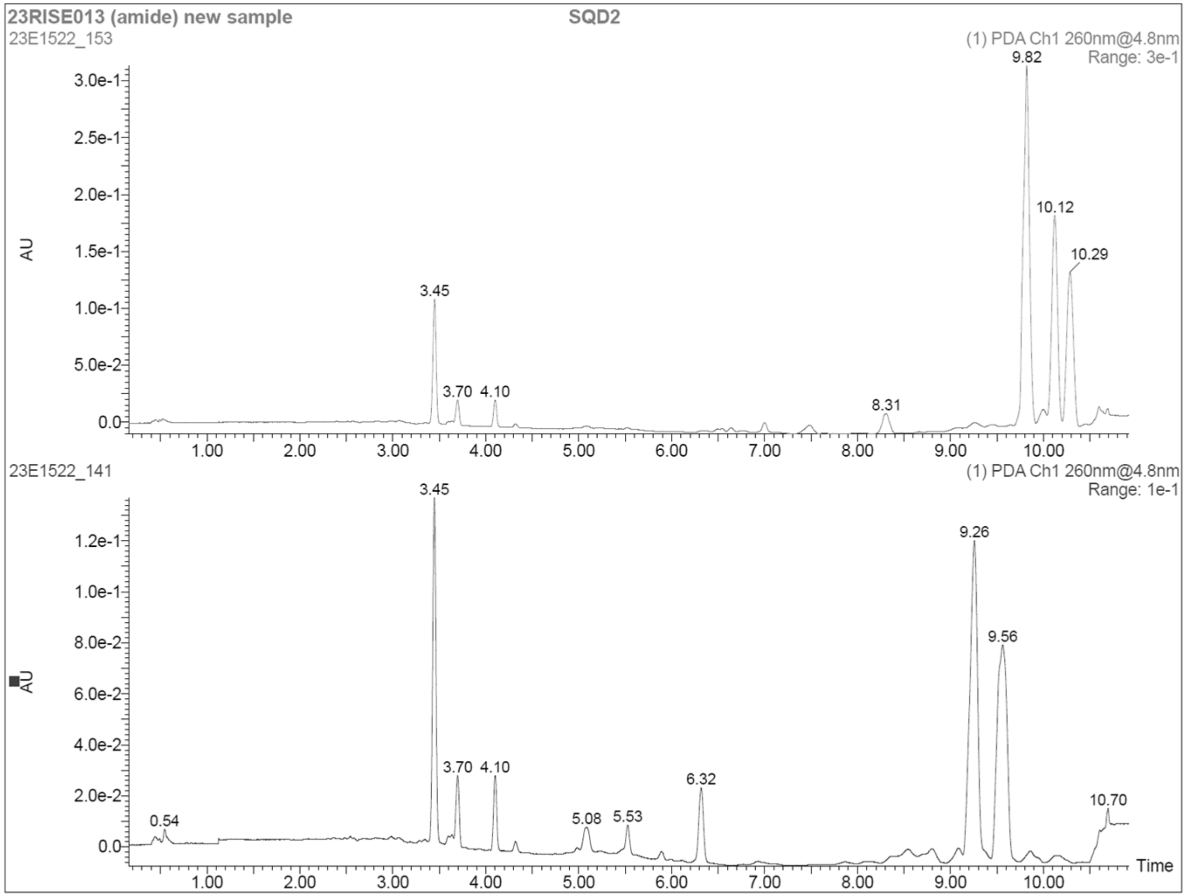

Figure S21. Comparison of HPLC profiles of $(B)_2$-T_5 before and after SPAAC. Trityl (DMT) is intact in both scenarios. Above: RP-HPLC profile of $(B)_2$-T_5 + Fmoc-L-Lys(N_3)-OH (DMT-on), 9.82 min-azide, 10.0 min: monoconjugate.10.12 and 10.29 min: bis-conjugate after 3 h reaction time. Bellow: RP-HPLC profile of $(B)_2$-T_5, 9.26 and 9.56 min: $(B)_2$-T_5 (DMT-on, both diastereomers). 0.4 ml/min gradient from 0 -60-80% MeOH in 8.6 mM TEA + 100 mM HFIP buffer over 15 min at 40 °C.

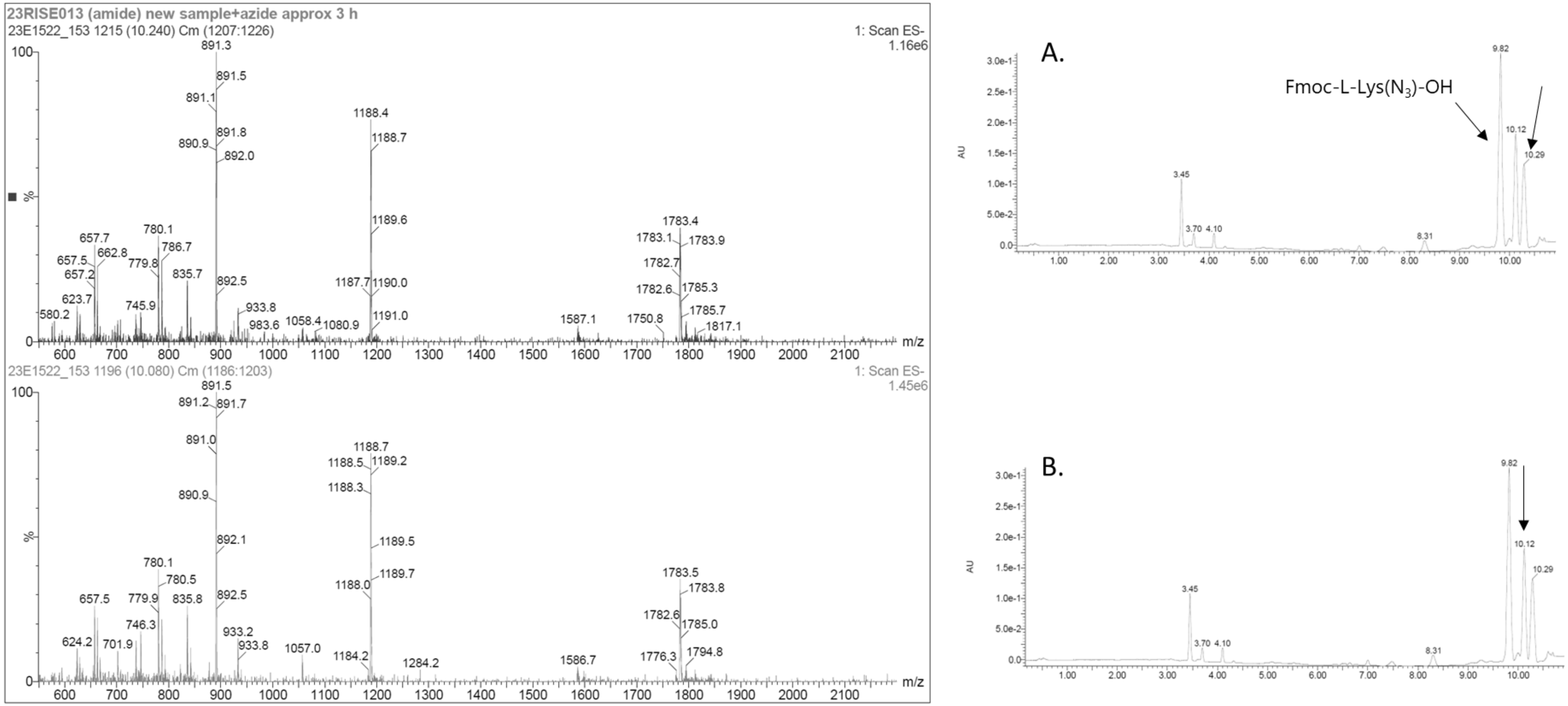

Figure S22. MS and RP-HPLC profile of $(B)_2$-T_5 after SPAAC ($(B)_2$-T_5 +Fmoc-L-Lys(N_3)-OH (DMT-on, both isomers)) (diastereomers are separating on HPLC because of phosphorothioate linkages). The arrows indicate on which isomer MS profile is shown. Mw$[M-3H]^{3-}$ calc. 1188.2, obs. 1188.4 and 1188.7. 0.4 ml/min gradient from 0 -60-80% MeOH in 8.6 mM TEA + 100 mM HFIP buffer over 15 min at 40°C.

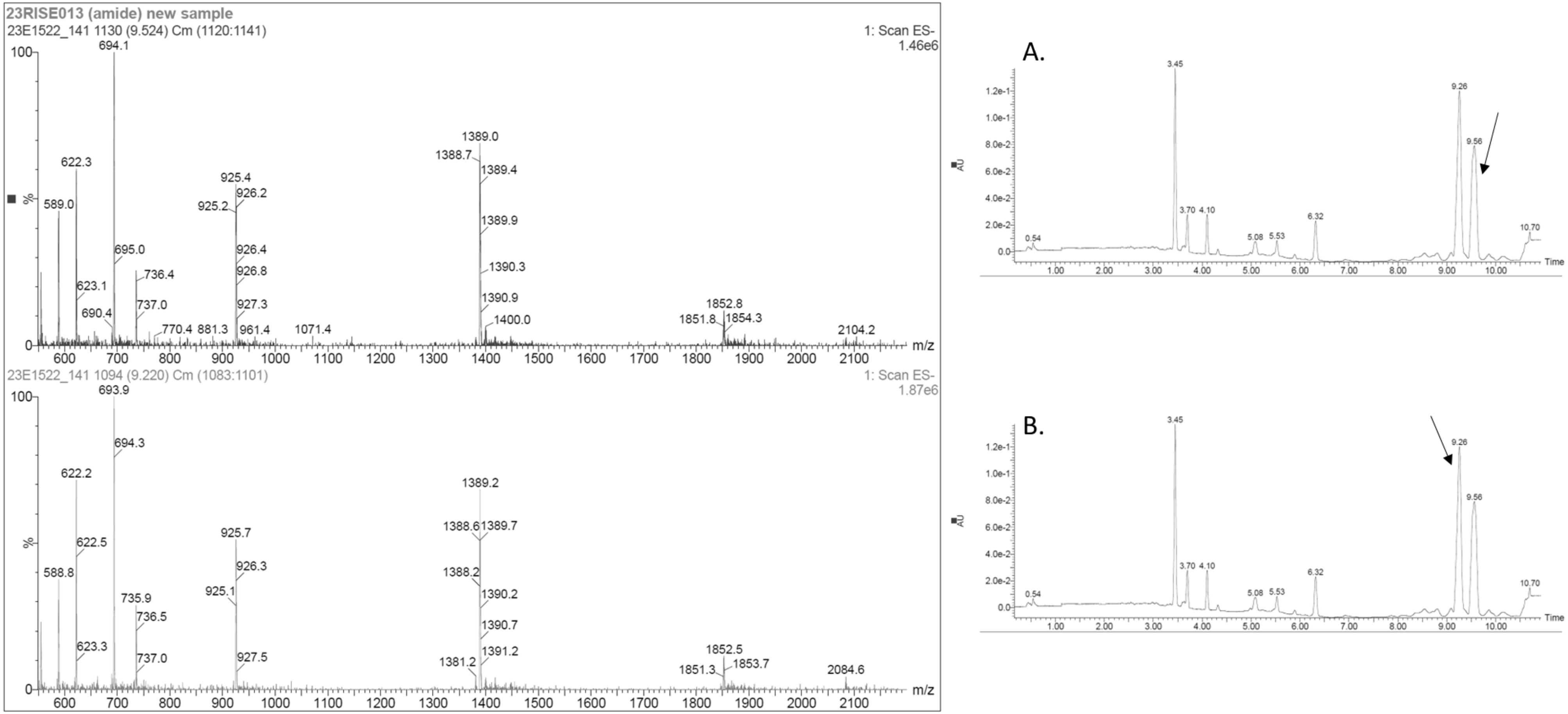

Figure S23. MS and RP-HPLC profile of (B)$_2$-T$_5$ (DMT on, both diastereomers before SPAAC). Mw[M-3H]$^{3-}$ calc. 925.2, obs. 925.4 and 925.7. 0.4 ml/min gradient from 0 -60-80% MeOH in 8.6 mM TEA + 100 mM HFIP buffer over 15 min at 40°C.

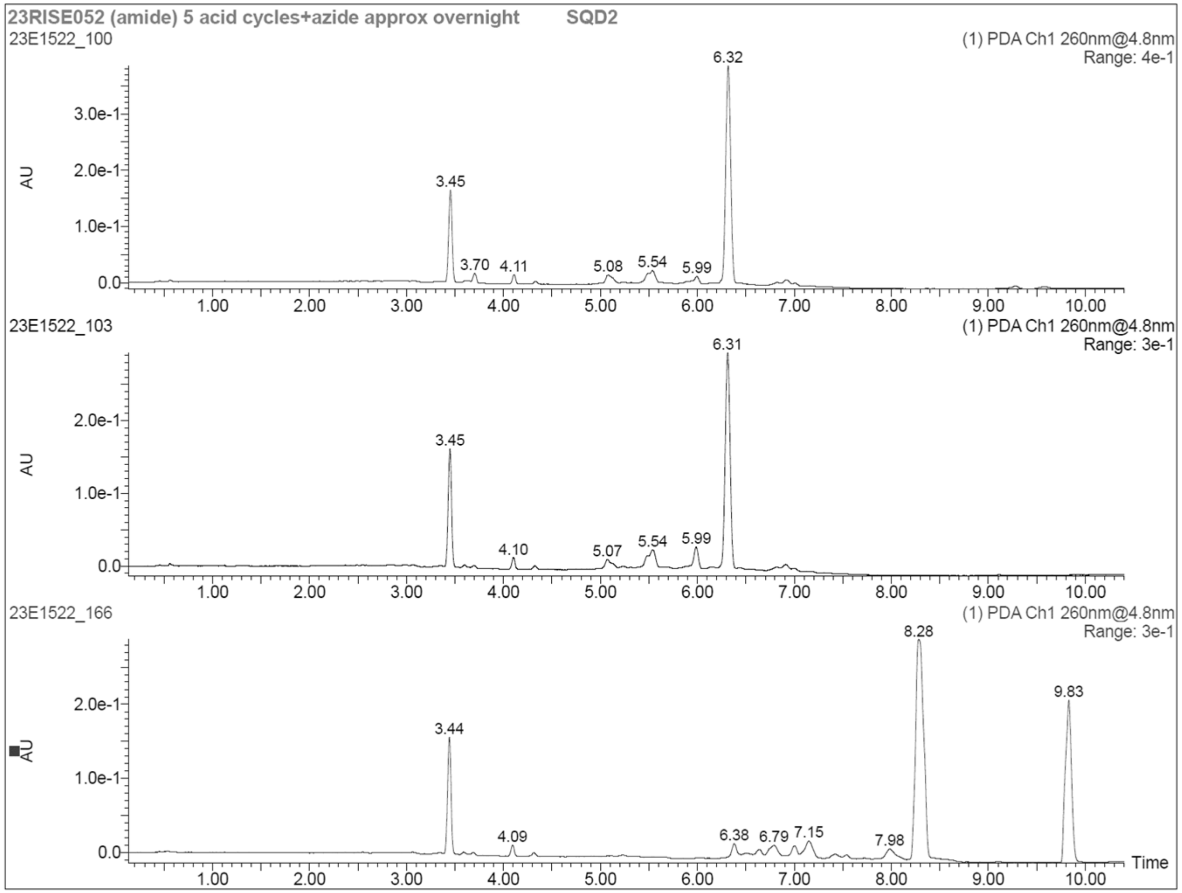

Figure S24. Comparison of RP-HPLC profiles of $(B)_2$-T_5 after different one, five detritylation cycles and after SPAAC reaction when the sample was subjected to five detritylation cycles. Above: RP-HPLC profile of $(B)_2$-T_5 after one detritylation cycle. Middle: RP-HPLC profile of $(B)_2$-T_5 after five detritylation cycles. 6.31 min: $(B)_2$-T_5, 5.9 min: $(B)_2$-T_5+H_2O, 5.54 min: $(B)_2$-T_5+2H_2O. Bellow: RP-HPLC profile of $(B)_2$-T_5 + Fmoc-L-Lys(N_3)-OH after reaction overnight at ambient temperature, 7.98 min: monoconjugate, 8.28: 0.4 ml/min gradient from 0 -60-80% MeOH in 8.6 mM TEA + 100 mM HFIP buffer over 15 min at 40°C.

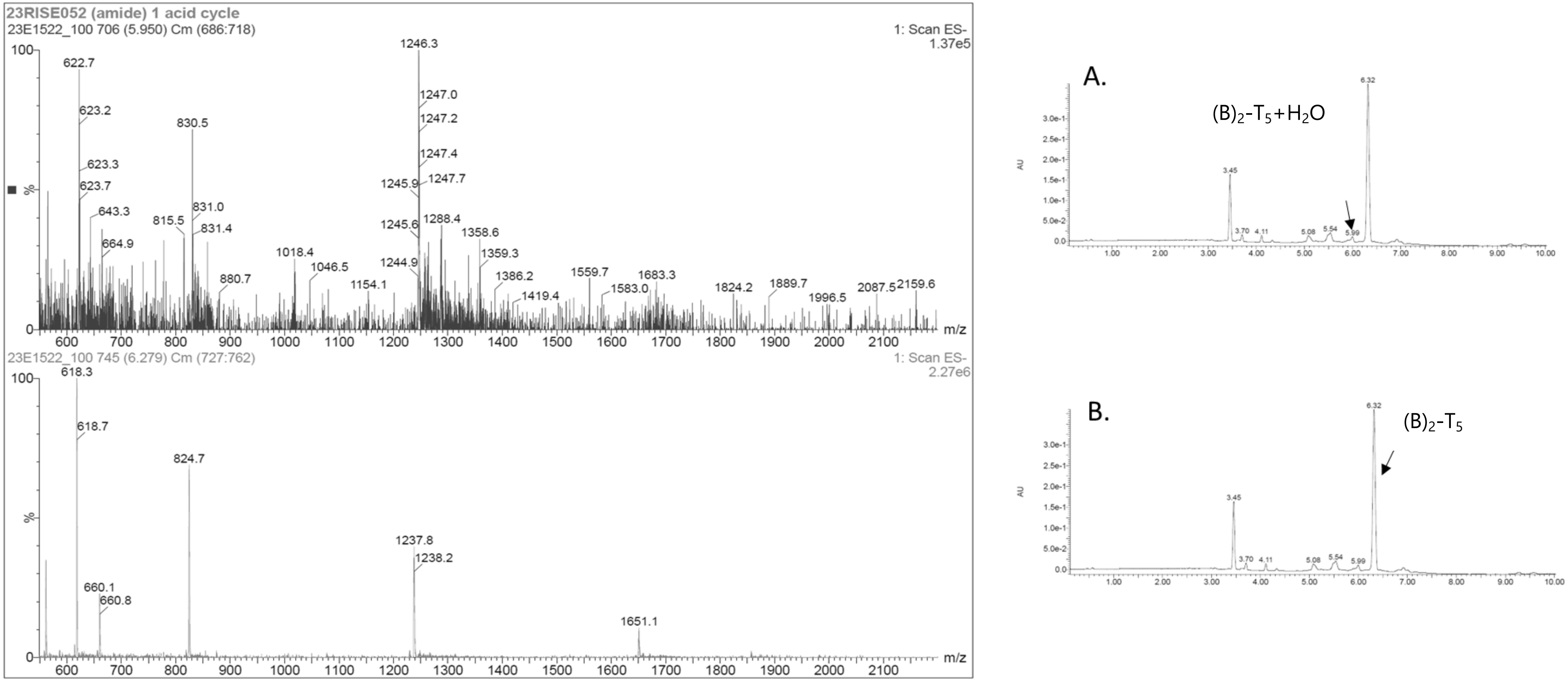

Figure S25. MS and RP-HPLC profile of (B)$_2$-T$_5$ after one detritylation cycle. A.: (B)$_2$-T$_5$+H$_2$O, Mw[M-2H]$^{2-}$ calc.1246.1, obs. 1246.3. B.: (B)$_2$-T$_5$, Mw[M-2H]$^{2-}$ calc.1237.8, obs. 1237.8.

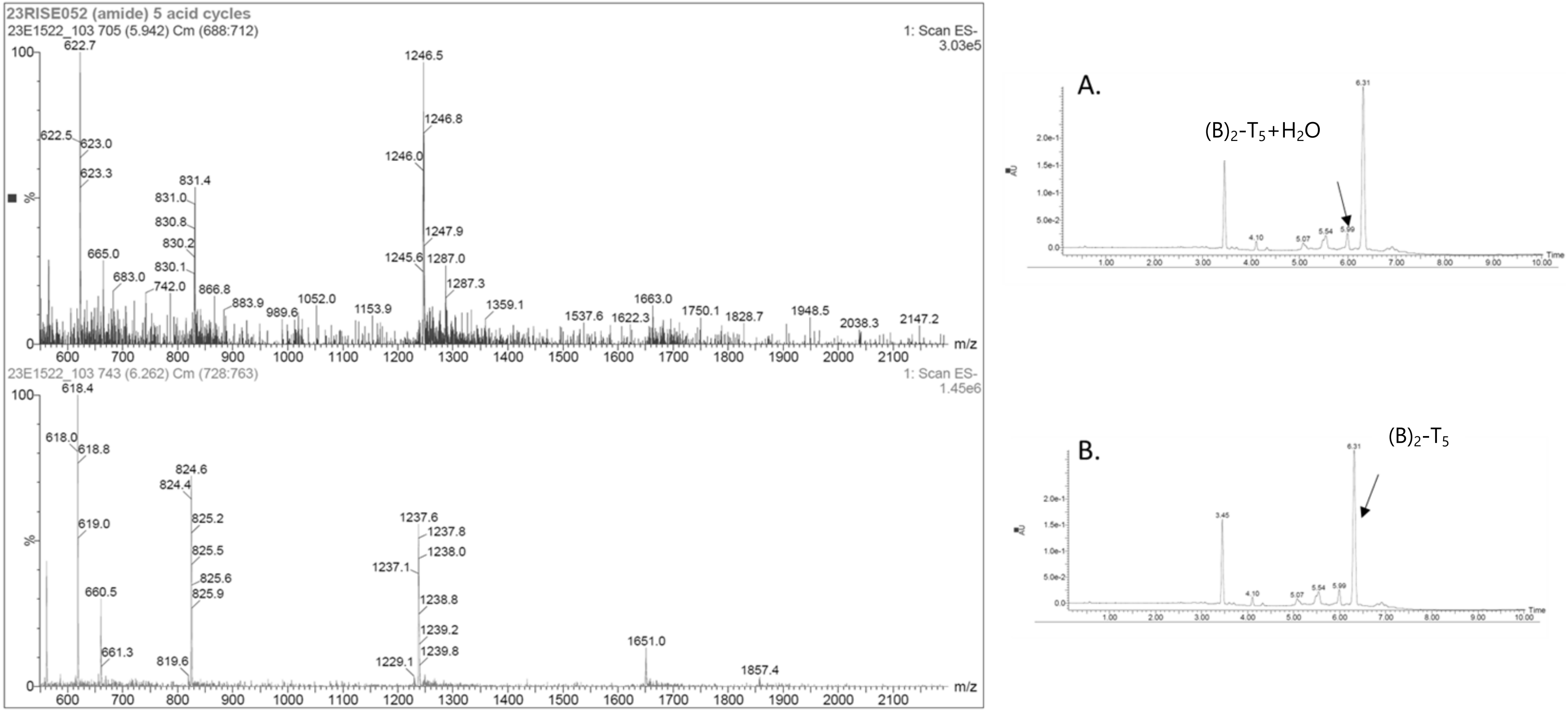

Figure S26. MS and HPLC profile of $(B)_2$-T_5 and RP-HPLC profiles after five detritylation cycles. A.: $(B)_2$-T_5+H_2O, Mw$[M-2H]^{2-}$ calc.1246.1, obs. 1246.5. B.: $(B)_2$-T_5, Mw$[M-2H]^{2-}$ calc.1237.8, obs. 1237.6.

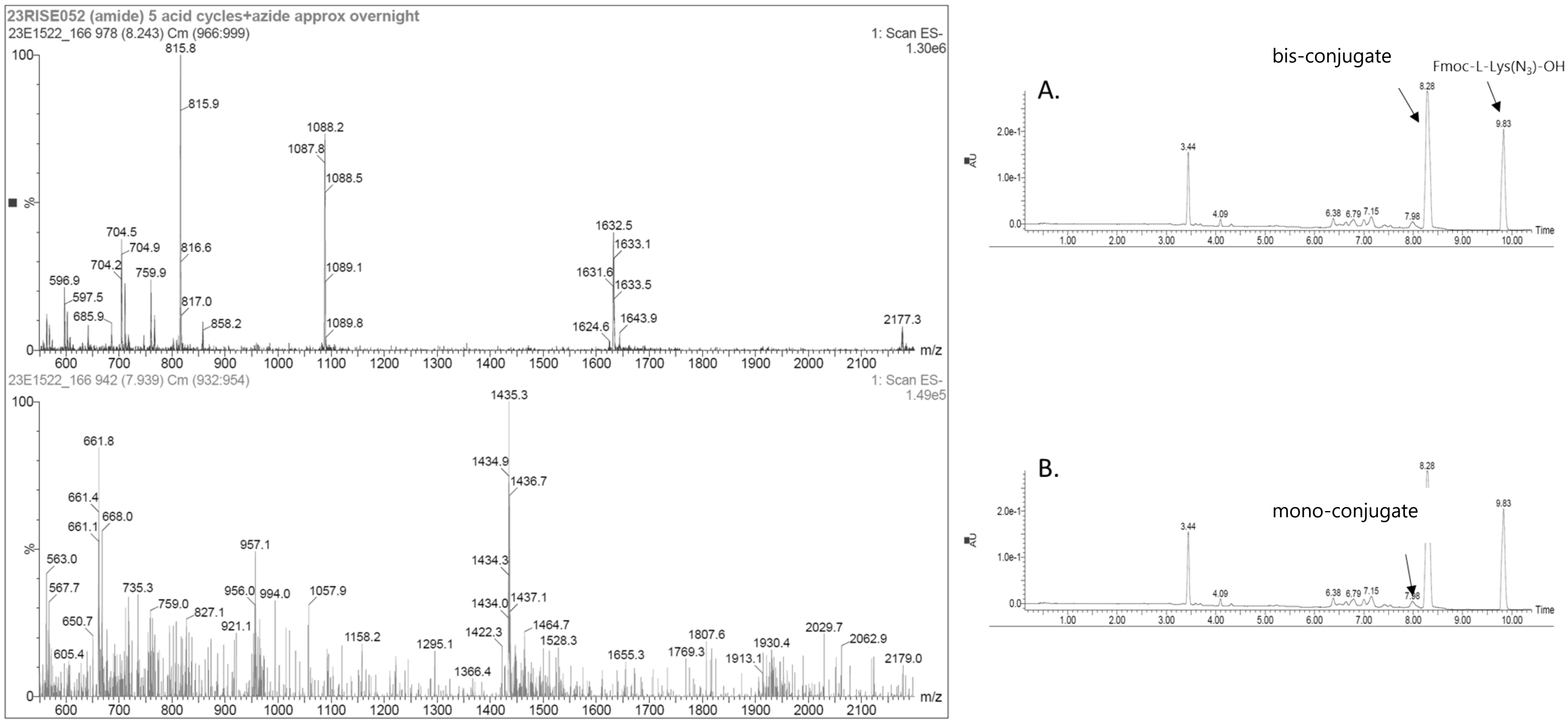

Figure S27. MS and RP-HPLC profile of $(B)_2$-T_5 after five detritylation cycles + Fmoc-L-Lys(N3)-OH. A.: Mw$[M-2H]^{2-}$ calc. 1632.1, obs. 1632.5. B. Mw$[M-2H]^{2-}$ calc. 1434.4, obs. 1435.3.

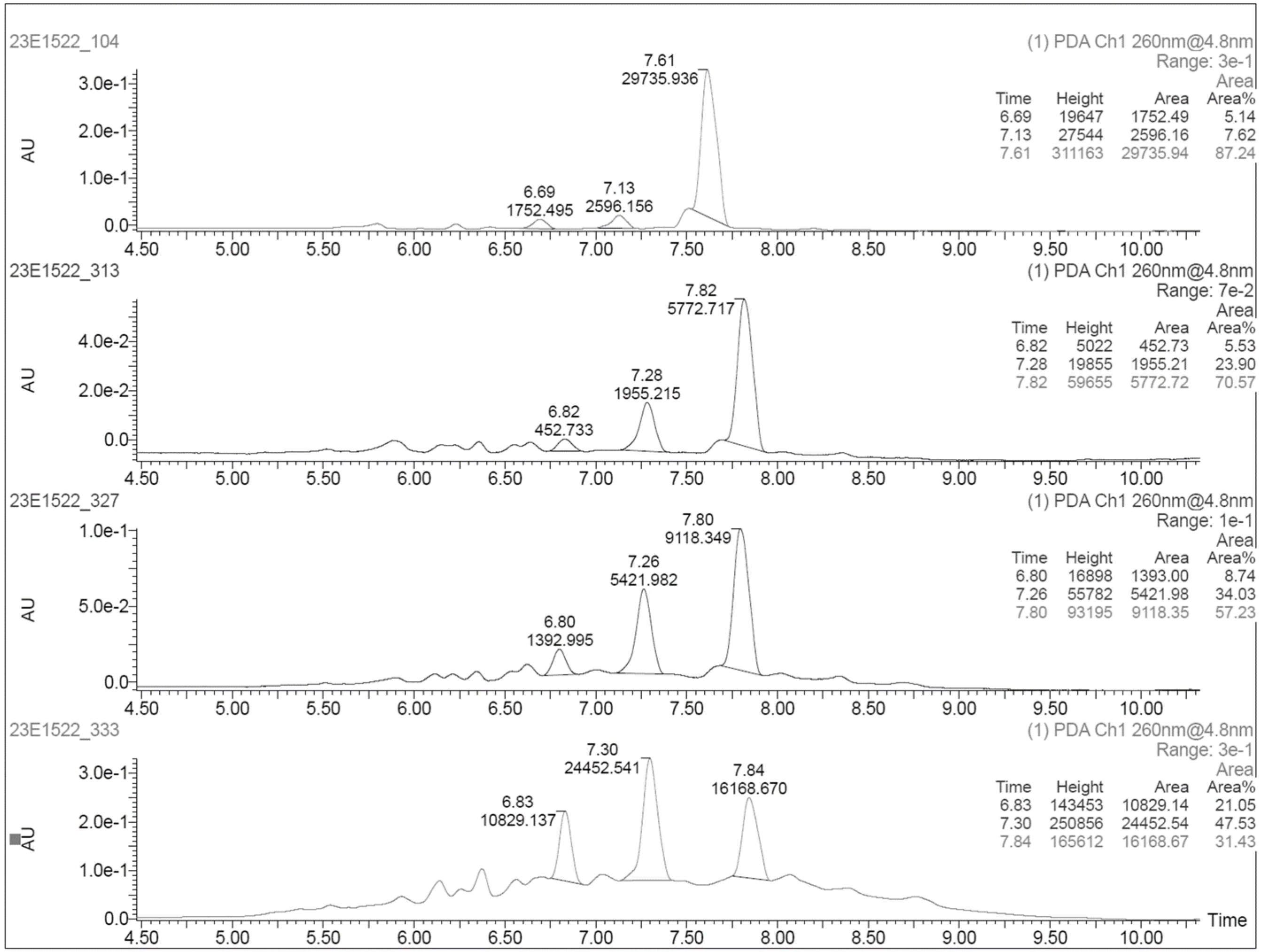

Figure S28. From above: RP-HPLC profiles of (A)$_2$-T$_5$ after 1, 5, 10 and 20 detritylation cycles.

A.

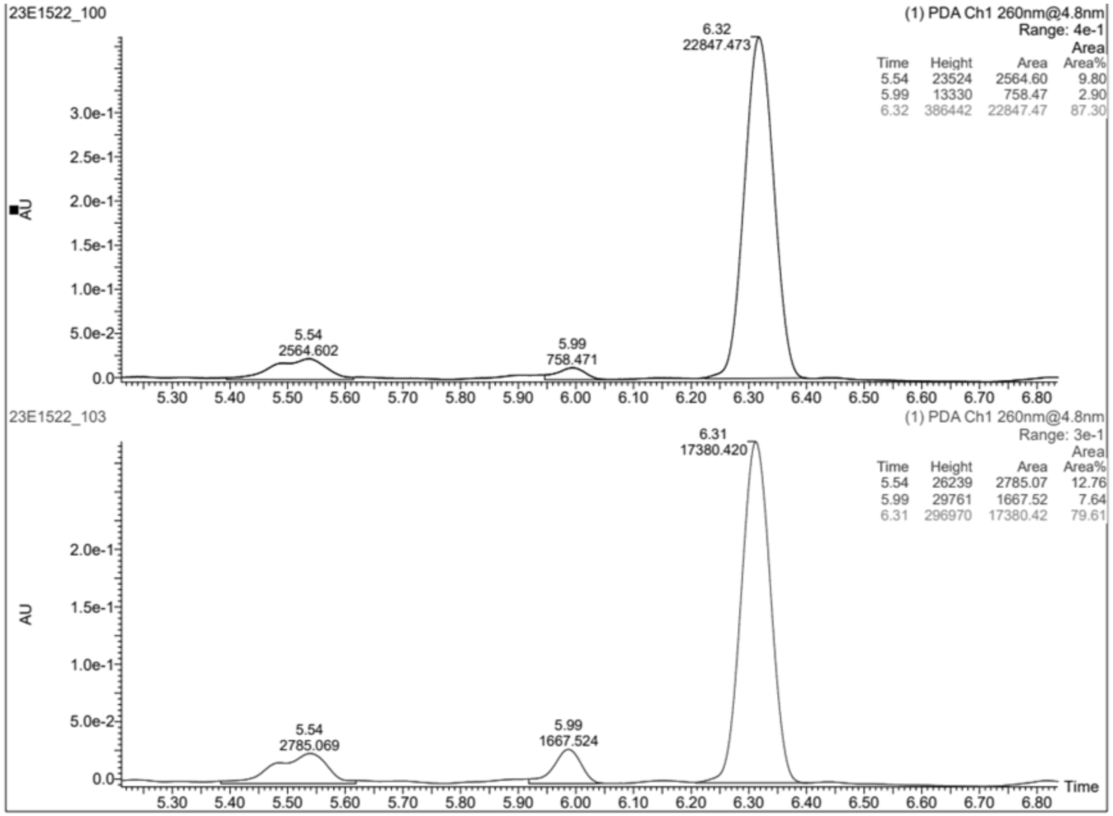

B.

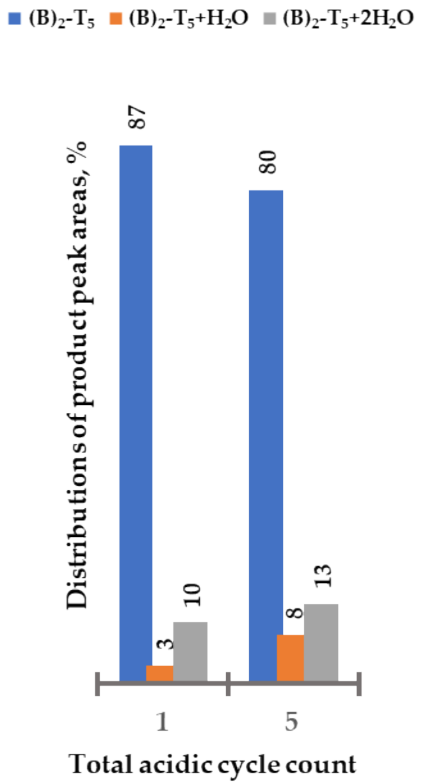

Figure S29. A.; From above: RP-HPLC profiles of $(B)_2$-T_5 after 1 and 5 detritylation cycles. B.: Distribution of $(B)_2$-T_5 and side products ($(B)_2$-T_5 + H_2O and $(B)_2$-T_5 + $2H_2O$) after 1 and 5 of standard detritylation treatments in percent of total peak area.

1. Pieken, W.W., Andreas ; Sebesta, David P ; Leuck, Michael ; Latham-Timmons, Hallie A; Pilon, John; Husar, Gregory M;, *Method for immobilizing oligonucleotides employing the cycloaddition bioconjugation method*. 2001, USA.
